# Supplementary material for: Allosteric Role of Substrate Occupancy Toward the Alignment of P-glycoprotein Nucleotide Binding Domains
Source: Sci Rep. 2018 Oct 2;8:14643. doi: 10.1038/s41598-018-32815-2 (PMC6168518; doi:10.1038/s41598-018-32815-2)
Supplement: Supplementary file 1 — Supporting information [file 41598_2018_32815_MOESM1_ESM.pdf]

Allosteric Role of Substrate Occupancy Toward the Alignment of P-glycoprotein Nucleotide Binding Domains

Lurong Pan[1] and Stephen G Aller[2]*

[1,2]Department of Pharmacology and Toxicology, Center for Structural Biology, University of Alabama at Birmingham, 1025 18th Street South, Birmingham, AL, 35205

*To whom correspondence should be addressed: sgaller@uab.edu

(Supporting information)

Table S1. Docking results between various substrate and Pgp crystal structures

**Ligands**	**Receptor**	**Mean binding energy (Kcal/mol)**	**Clustering**
Atorvastatin	4M1M(A)	-8.66	3
	4M1M(B)	-7.82	2
	4M2S(A)	-10.15	2
	4M2S(B)	-9.3	2
	4M2T(A)	-9.3	2
	4M2T(B)	-9.56	2
Chloroquine	4M1M(A)	-6.75	9
	4M1M(B)	-7.09	20
	4M2S(A)	-7.28	17
	4M2S(B)	-7.4	6
	4M2T(A)	-6.84	2
	4M2T(B)	-6.64	2
Ciprofloxacin	4M1M(A)	-6.75	32
	4M1M(B)	-6.59	39
	4M2S(A)	-6.78	88
	4M2S(B)	-6.88	16
	4M2T(A)	-6.4	37
	4M2T(B)	-7.01	28
Colchicine	4M1M(A)	-7.91	6
	4M1M(B)	-8.1	43
	4M2S(A)	-8.58	16
	4M2S(B)	-8.55	17
	4M2T(A)	-7.8	10

	4M2T(B)	-7.7	4
Cyclosporine	4M1M(A)	-0.32	62
	4M1M(B)	24.91	54
	4M2S(A)	-2.81	76
	4M2S(B)	4.8	80
	4M2T(A)	1.62	14
	4M2T(B)	7.34	14
Daunorubicin	4M1M(A)	-8.65	20
	4M1M(B)	-9.97	5
	4M2S(A)	-8.83	9
	4M2S(B)	-9.94	27
	4M2T(A)	-8.97	4
	4M2T(B)	-9.54	3
Digitoxin	4M1M(A)	-10.36	4
	4M1M(B)	-9.43	5
	4M2S(A)	-10.78	3
	4M2S(B)	-9.84	7
	4M2T(A)	-10.53	2
	4M2T(B)	-10.42	7
Digoxin	4M1M(A)	-10.65	9
	4M1M(B)	-11.04	4
	4M2S(A)	-11.74	3
	4M2S(B)	-9.85	2
	4M2T(A)	-10.84	2
	4M2T(B)	-10.28	3
Docetaxel	4M1M(A)	-9.35	2
	4M1M(B)	-8.16	3
	4M2S(A)	-9.14	2
	4M2S(B)	-7.82	7
	4M2T(A)	-10.36	3
	4M2T(B)	-8.19	5
Doxorubicin	4M1M(A)	-9.31	17
	4M1M(B)	-9.3	10
	4M2S(A)	-9.74	4
	4M2S(B)	-10.12	19
	4M2T(A)	-9.48	3
	4M2T(B)	-9.46	4
Epirubicin	4M1M(A)	-9.47	10
	4M1M(B)	-8.9	7
	4M2S(A)	-10.1	9
	4M2S(B)	-10.03	2
	4M2T(A)	-9.29	2

	4M2T(B)	-9.81	5
Estradiol	4M1M(A)	-7.51	16
	4M1M(B)	-7.48	42
	4M2S(A)	-8.16	7
	4M2S(B)	-8.21	34
	4M2T(A)	-7.15	6
	4M2T(B)	-7.69	17
Etoposide	4M1M(A)	-9.47	23
	4M1M(B)	-9.49	12
	**4M2S(A)**	**-10.31**	**47**
	4M2S(B)	-9.79	9
	4M2T(A)	-9.6	9
	4M2T(B)	-10.25	11
Imatinib	4M1M(A)	-8.74	18
	4M1M(B)	-9.19	2
	4M2S(A)	-10.23	2
	4M2S(B)	-10.12	44
	4M2T(A)	-8.74	16
	4M2T(B)	-9.07	27
Indinavir	4M1M(A)	-8.94	2
	4M1M(B)	-8.02	6
	4M2S(A)	-9.32	2
	4M2S(B)	-9.12	2
	4M2T(A)	-9.06	2
	4M2T(B)	-9.45	2
Irinotecan	4M1M(A)	-11.56	25
	4M1M(B)	-11.05	9
	4M2S(A)	-10.8	2
	**4M2S(B)**	**-12.45**	**17**
	4M2T(A)	-12.21	3
	4M2T(B)	-11.57	2
Ivermectin	4M1M(A)	-7.92	2
	4M1M(B)	-6.38	6
	4M2S(A)	-7.6	7
	4M2S(B)	-7.01	15
	4M2T(A)	-9.18	25
	4M2T(B)	-6.85	4
Mitoxantrone	4M1M(A)	-8.35	6
	4M1M(B)	-7.63	8
	4M2S(A)	-8.95	16
	4M2S(B)	-9.3	3
	4M2T(A)	-7.8	11

	4M2T(B)	-7.83	2
Nicardipine	4M1M(A)	-8.17	2
	4M1M(B)	-8.54	2
	4M2S(A)	-9.5	5
	4M2S(B)	-8.85	2
	4M2T(A)	-8.23	8
	4M2T(B)	-8.8	8
Paclitaxel	4M1M(A)	-8.87	3
	4M1M(B)	-7.42	7
	4M2S(A)	-9.32	2
	4M2S(B)	-6.52	3
	4M2T(A)	-10.19	2
	4M2T(B)	-8.54	4
Progesterone	4M1M(A)	-8.73	86
	4M1M(B)	-8.47	96
	4M2S(A)	-8.51	46
	4M2S(B)	-9.34	80
	4M2T(A)	-8.52	99
	4M2T(B)	-9.04	86
Quinidine	4M1M(A)	-7.3	3
	4M1M(B)	-7.21	7
	4M2S(A)	-7.88	41
	4M2S(B)	-7.72	21
	4M2T(A)	-7.19	66
	4M2T(B)	-7.37	3
Ritonavir	4M1M(A)	-8.62	2
	4M1M(B)	-7.18	2
	4M2S(A)	-10.22	1
	4M2S(B)	-9.89	1
	4M2T(A)	-9.34	2
	4M2T(B)	-7.25	2
Saquinavir	4M1M(A)	-9.88	2
	4M1M(B)	-9.15	2
	4M2S(A)	-11.14	2
	4M2S(B)	-10.36	2
	4M2T(A)	-10.54	2
	4M2T(B)	-10.3	2
Tamoxifen	4M1M(A)	-7.88	3
	4M1M(B)	-7.63	7
	4M2S(A)	-8.4	13
	4M2S(B)	-8.19	49
	4M2T(A)	-7.44	5

	4M2T(B)	-7.93	4
Tetracycline	4M1M(A)	-8.33	6
	4M1M(B)	-8.4	49
	4M2S(A)	-8.69	11
	4M2S(B)	-7.62	15
	4M2T(A)	-6.87	10
	4M2T(B)	-7.21	12
Topotecan	4M1M(A)	-7.44	18
	4M1M(B)	-7.71	4
	4M2S(A)	-8.15	4
	4M2S(B)	-7.99	31
	4M2T(A)	-7.85	19
	4M2T(B)	-6.92	47
Verapamil	4M1M(A)	-7.05	2
	4M1M(B)	-6.52	2
	4M2S(A)	-7.29	2
	4M2S(B)	-7.16	5
	4M2T(A)	-6.53	2
	4M2T(B)	-6.89	4
Vinblastine	**4M1M(A)**	**-11.38**	**14**
	4M1M(B)	-9.5	10
	4M2S(A)	-11.89	40
	4M2S(B)	-10.07	20
	4M2T(A)	-11.27	35
	4M2T(B)	-9.64	21
Vincristine	4M1M(A)	-9.07	21
	4M1M(B)	-6.22	2
	4M2S(A)	-10.34	39
	4M2S(B)	-8.33	22
	4M2T(A)	-8.73	21
	4M2T(B)	-8.74	2
Vinorelbine	4M1M(A)	-10.39	2
	4M1M(B)	-8.09	2
	4M2S(A)	-10.47	33
	4M2S(B)	-7.28	17
	4M2T(A)	-10.39	2
	4M2T(B)	-8.56	7

Table S2. Force Constants

1. cyclic-tris-(R)-valineselenazole (QZ59-RRR)

PSF file

```
66 !NATOM
1 L 1 LIG C4 C5B 0.105877 12.0110 0
2 L 1 LIG C8 C5A 0.060057 12.0110 0
3 L 1 LIG C9 C5A -0.290251 12.0110 0
4 L 1 LIG C10 C5B -0.151030 12.0110 0
5 L 1 LIG C14 C5A -0.167340 12.0110 0
6 L 1 LIG C15 C5A -0.309884 12.0110 0
7 L 1 LIG C16 C5B 0.041544 12.0110 0
8 L 1 LIG C20 C5A -0.167545 12.0110 0
9 L 1 LIG C21 C5A -0.343736 12.0110 0
10 L 1 LIG N22 N5B -0.337007 14.0067 0
11 L 1 LIG N23 N5B -0.144268 14.0067 0
12 L 1 LIG N24 N5B -0.090404 14.0067 0
13 L 1 LIG S1 SE 0.248629 78.9600 0
14 L 1 LIG S2 SE 0.230506 78.9600 0
15 L 1 LIG S3 SE 0.268015 78.9600 0
16 L 1 LIG C5 C=O 0.740864 12.0110 0
17 L 1 LIG N6 NC=O -0.423776 14.0067 0
18 L 1 LIG C7 CR -0.239462 12.0110 0
19 L 1 LIG C11 C=O 0.885337 12.0110 0
20 L 1 LIG N12 NC=O -0.793706 14.0067 0
21 L 1 LIG C13 CR 0.508120 12.0110 0
22 L 1 LIG C17 C=O 0.557654 12.0110 0
23 L 1 LIG N18 NC=O -0.442706 14.0067 0
24 L 1 LIG C19 CR 0.709255 12.0110 0
25 L 1 LIG O25 O=C -0.648647 15.9994 0
26 L 1 LIG O26 O=C -0.646564 15.9994 0
27 L 1 LIG O27 O=C -0.618095 15.9994 0
28 L 1 LIG C28 CR 0.315834 12.0110 0
29 L 1 LIG C29 CR -0.097642 12.0110 0
30 L 1 LIG C30 CR -0.115963 12.0110 0
31 L 1 LIG C31 CR 0.609719 12.0110 0
32 L 1 LIG C32 CR -0.181323 12.0110 0
33 L 1 LIG C33 CR -0.334350 12.0110 0
34 L 1 LIG C34 CR 0.101079 12.0110 0
35 L 1 LIG C35 CR -0.142577 12.0110 0
36 L 1 LIG C36 CR -0.363155 12.0110 0
37 L 1 LIG H37 HNCO 0.205514 1.0079 0
38 L 1 LIG H38 HCMM 0.208999 1.0079 0
39 L 1 LIG H39 HCMM 0.201224 1.0079 0
40 L 1 LIG H40 HNCO 0.232969 1.0079 0
41 L 1 LIG H41 HCMM -0.024783 1.0079 0
42 L 1 LIG H42 HCMM 0.212352 1.0079 0
```

43	L	1	LIG	H43	HNCO	0.146657	1.0079	0
44	L	1	LIG	H44	HCMM	-0.031987	1.0079	0
45	L	1	LIG	H45	HCMM	0.205138	1.0079	0
46	L	1	LIG	H46	HCMM	-0.079776	1.0079	0
47	L	1	LIG	H47	HCMM	0.013063	1.0079	0
48	L	1	LIG	H48	HCMM	0.023588	1.0079	0
49	L	1	LIG	H49	HCMM	0.001499	1.0079	0
50	L	1	LIG	H50	HCMM	0.024831	1.0079	0
51	L	1	LIG	H51	HCMM	0.030798	1.0079	0
52	L	1	LIG	H52	HCMM	0.020932	1.0079	0
53	L	1	LIG	H53	HCMM	-0.237712	1.0079	0
54	L	1	LIG	H54	HCMM	0.010879	1.0079	0
55	L	1	LIG	H55	HCMM	0.019222	1.0079	0
56	L	1	LIG	H56	HCMM	0.012655	1.0079	0
57	L	1	LIG	H57	HCMM	0.062713	1.0079	0
58	L	1	LIG	H58	HCMM	0.058301	1.0079	0
59	L	1	LIG	H59	HCMM	0.054409	1.0079	0
60	L	1	LIG	H60	HCMM	0.012363	1.0079	0
61	L	1	LIG	H61	HCMM	0.037354	1.0079	0
62	L	1	LIG	H62	HCMM	0.033099	1.0079	0
63	L	1	LIG	H63	HCMM	-0.005997	1.0079	0
64	L	1	LIG	H64	HCMM	0.029770	1.0079	0
65	L	1	LIG	H65	HCMM	0.124987	1.0079	0
66	L	1	LIG	H66	HCMM	0.063875	1.0079	0

BONDS

C5B	C=O	380.558	1.4310
C5B	C5A	512.256	1.3770
C5B	N5B	320.682	1.3690
C=O	NC=O	419.491	1.3690
C=O	O=C	931.963	1.2220
CR	C5A	322.481	1.4710
CR	CR	306.432	1.5080
CR	HCMM	342.991	1.0930
CR	NC=O	335.651	1.4360
C5A	N5B	599.191	1.3130
C5A	SE	182.039	1.878
C5A	HCMM	398.045	1.0800
HNCO	NC=O	479.511	1.0150

ANGLES

C5A	C5B	N5B	74.700	111.6210
C5A	C5B	C=O	59.588	124.8900
N5B	C5B	C=O	68.295	121.8210
N5B	C5A	SE	32.227	113.5700
N5B	C5A	CR	62.250	127.6100
SE	C5A	CR	27.131	121.6367
C5B	C5A	SE	27.000	110.0200
C5B	C5A	HCMM	41.524	131.7210

SE C5A HCMM   19.399   123.886
C5B N5B C5A   86.791   103.7790
C5A SE C5A   25.211   85.0166
C5B C=O NC=O   79.018   113.2330
C5B C=O O=C   77.075   124.1330
NC=O C=O O=C   65.273   127.1520
C=O NC=O CR   59.084   119.6000
C=O NC=O HNCO   41.380   120.2770
CR   NC=O HNCO   39.725   120.0660
C5A CR   NC=O   71.966   109.5000
C5A CR   CR   72.397   110.0580
C5A CR   HCMM   44.691   110.4670
NC=O CR   CR   75.564   109.9600
NC=O CR   HCMM   53.255   107.6460
CR   CR   HCMM   45.770   110.5490
CR   CR   CR   61.243   109.6080
HCMM CR   HCMM   37.134   108.8360

DIHEDRALS
C5B C5A SE C5A   3.500 2   180.00
C5B N5B C5A SE   3.500 2   180.00
C5B N5B C5A CR   3.500 2   180.00
C5B C=O NC=O CR   3.000 2   180.00
C5B C=O NC=O HNCO   3.000 2   180.00
C5A N5B C5B C5A   3.500 2   180.00
C5A N5B C5B C=O   3.500 2   180.00
C5A SE C5A HCMM   3.500 2   180.00
C5A CR   NC=O C=O   0.500 3   0.00
C5A CR   NC=O HNCO   0.150 3   0.00
C5A CR   CR   CR   0.150 3   0.00
C5A CR   CR   HCMM   0.150 3   0.00
C5A C5B C=O NC=O   1.250 2   180.00
C5A C5B C=O O=C   1.250 2   180.00
C5A SE C5A N5B   3.500 2   180.00
C5A SE C5A CR   3.500 2   180.00
N5B C5B C5A SE   3.500 2   180.00
N5B C5B C5A HCMM   3.500 2   180.00
N5B C5B C=O NC=O   1.250 2   180.00
N5B C5B C=O O=C   1.250 2   180.00
N5B C5A CR   NC=O   0.000 1   0.00
N5B C5A CR   CR   0.000 1   0.00
N5B C5A CR   HCMM   0.000 1   0.00
SE C5A CR   NC=O   0.000 1   0.00
SE C5A CR   CR   0.000 1   0.00
SE C5A CR   HCMM   0.000 1   0.00
SE C5A C5B C=O   3.500 2   180.00
C=O C5B C5A HCMM   0.000 1   0.00
C=O NC=O CR   CR   -0.513 1   0.00

```
C=O  NC=O CR   CR       0.347  2   180.00
C=O  NC=O CR   CR       0.474  3     0.00
C=O  NC=O CR   HCMM    -1.050  1     0.00
C=O  NC=O CR   HCMM     0.681  2   180.00
C=O  NC=O CR   HCMM     0.011  3     0.00
NC=O CR   CR   CR       0.150  3     0.00
NC=O CR   CR   HCMM     0.213  3     0.00
CR   NC=O C=O  O=C     -0.160  1     0.00
CR   NC=O C=O  O=C      3.147  2   180.00
CR   NC=O C=O  O=C     -0.073  3     0.00
CR   CR   CR   HCMM     0.320  1     0.00
CR   CR   CR   HCMM    -0.315  2   180.00
CR   CR   CR   HCMM     0.132  3     0.00
O=C  C=O  NC=O HNCO     0.718  1     0.00
O=C  C=O  NC=O HNCO     2.487  2   180.00
O=C  C=O  NC=O HNCO    -0.227  3     0.00
CR   CR   NC=O HNCO     0.276  1     0.00
CR   CR   NC=O HNCO    -0.190  2   180.00
CR   CR   NC=O HNCO     0.163  3     0.00
HNCO NC=O CR   HCMM    -0.308  1     0.00
HNCO NC=O CR   HCMM     0.137  3     0.00
HCMM CR   CR   HCMM     0.142  1     0.00
HCMM CR   CR   HCMM    -0.693  2   180.00
HCMM CR   CR   HCMM     0.157  3     0.00
```

IMPROPER

```
C5B  N5B  C=O  C5A      2.879  0     0.00
C5A  CR   N5B  SE       3.598  0     0.00
C=O  NC=O C5B  O=C      9.356  0     0.00
NC=O CR   C=O  HNCO    -1.439  0     0.00
CR   C5A  NC=O CR       0.000  0     0.00
CR   CR   NC=O HCMM     0.000  0     0.00
C5A  N5B  CR   SE       3.598  0     0.00
C5A  C5B  SE HCMM       1.008  0     0.00
C5B  N5B  C5A  C=O      2.879  0     0.00
CR   C5A  NC=O HCMM     0.000  0     0.00
C=O  O=C  C5B  NC=O     9.356  0     0.00
CR   CR   CR   CR       0.000  0     0.00
CR   CR   CR   HCMM     0.000  0     0.00
CR   CR   C5A  HCMM     0.000  0     0.00
C5A  SE C5B  HCMM       1.008  0     0.00
CR   HCMM CR   HCMM     0.000  0     0.00
```

NONBONDED nbxmod 5 atom cdiel shift vatom vdistance vswitch -
cutnb 14.0 ctofnb 12.0 ctonnb 10.0 eps 1.0 e14fac 1.0 wmin 1.5

```
C5B     0.000000  -0.050000     2.040000
C5A     0.000000  -0.050000     2.040000
N5B     0.000000  -0.200000     1.850000
```

```
SE    0.000000  -0.0130000     1.669500
C=O     0.000000  -0.110000     2.000000
NC=O    0.000000  -0.200000     1.850000
CR      0.000000  -0.055000     2.175000   0.000000  -0.010000     1.900000
O=C     0.000000  -0.120000     1.700000   0.000000  -0.120000     1.400000
HCMM    0.000000  -0.022000     1.320000
HNCO    0.000000  -0.046000     0.224500
```

2. cyclic-tris-(R)-valinethiazole (QZ59-RRR(S))

PSF file

```
    66 !NATOM
     1 LIG  1    LIG  C    C5B    0.141200      12.0110          0
     2 LIG  1    LIG  C3   C5A    0.198100      12.0110          0
     3 LIG  1    LIG  C4   C5A   -0.110000      12.0110          0
     4 LIG  1    LIG  C5   C5B    0.141200      12.0110          0
     5 LIG  1    LIG  C8   C5A    0.198100      12.0110          0
     6 LIG  1    LIG  C9   C5A   -0.110000      12.0110          0
     7 LIG  1    LIG  C10  C5B    0.141200      12.0110          0
     8 LIG  1    LIG  C13  C5A    0.198100      12.0110          0
     9 LIG  1    LIG  C14  C5A   -0.110000      12.0110          0
    10 LIG  1    LIG  N3   N5B   -0.565300      14.0067          0
    11 LIG  1    LIG  N4   N5B   -0.565300      14.0067          0
    12 LIG  1    LIG  N5   N5B   -0.565300      14.0067          0
    13 LIG  1    LIG  S    STHI  -0.080000      32.0660          0
    14 LIG  1    LIG  S1   STHI  -0.080000      32.0660          0
    15 LIG  1    LIG  S2   STHI  -0.080000      32.0660          0
    16 LIG  1    LIG  C1   C=O    0.716000      12.0110          0
    17 LIG  1    LIG  N    NC=O  -0.730100      14.0067          0
    18 LIG  1    LIG  C2   CR     0.480100      12.0110          0
    19 LIG  1    LIG  C6   C=O    0.716000      12.0110          0
    20 LIG  1    LIG  N1   NC=O  -0.730100      14.0067          0
    21 LIG  1    LIG  C7   CR     0.480100      12.0110          0
    22 LIG  1    LIG  C11  C=O    0.716000      12.0110          0
    23 LIG  1    LIG  N2   NC=O  -0.730100      14.0067          0
    24 LIG  1    LIG  C12  CR     0.480100      12.0110          0
    25 LIG  1    LIG  O    O=C   -0.570000      15.9994          0
    26 LIG  1    LIG  O1   O=C   -0.570000      15.9994          0
    27 LIG  1    LIG  O2   O=C   -0.570000      15.9994          0
    28 LIG  1    LIG  C15  CR     0.000000      12.0110          0
    29 LIG  1    LIG  C16  CR     0.000000      12.0110          0
    30 LIG  1    LIG  C17  CR     0.000000      12.0110          0
    31 LIG  1    LIG  C18  CR     0.000000      12.0110          0
    32 LIG  1    LIG  C19  CR     0.000000      12.0110          0
    33 LIG  1    LIG  C20  CR     0.000000      12.0110          0
    34 LIG  1    LIG  C21  CR     0.000000      12.0110          0
    35 LIG  1    LIG  C22  CR     0.000000      12.0110          0
    36 LIG  1    LIG  C23  CR     0.000000      12.0110          0
    37 LIG  1    LIG  H    HCMM   0.000000       1.0079          0
    38 LIG  1    LIG  H1   HCMM   0.000000       1.0079          0
```

39	LIG	1	LIG	H2	HCMM	0.000000	1.0079	0
40	LIG	1	LIG	H3	HCMM	0.000000	1.0079	0
41	LIG	1	LIG	H4	HCMM	0.000000	1.0079	0
42	LIG	1	LIG	H5	HCMM	0.000000	1.0079	0
43	LIG	1	LIG	H6	HCMM	0.000000	1.0079	0
44	LIG	1	LIG	H7	HNCO	0.370000	1.0079	0
45	LIG	1	LIG	H8	HCMM	0.000000	1.0079	0
46	LIG	1	LIG	H9	HCMM	0.000000	1.0079	0
47	LIG	1	LIG	H10	HCMM	0.000000	1.0079	0
48	LIG	1	LIG	H11	HCMM	0.000000	1.0079	0
49	LIG	1	LIG	H12	HCMM	0.000000	1.0079	0
50	LIG	1	LIG	H13	HCMM	0.000000	1.0079	0
51	LIG	1	LIG	H14	HCMM	0.000000	1.0079	0
52	LIG	1	LIG	H15	HCMM	0.000000	1.0079	0
53	LIG	1	LIG	H16	HCMM	0.000000	1.0079	0
54	LIG	1	LIG	H17	HCMM	0.000000	1.0079	0
55	LIG	1	LIG	H18	HCMM	0.000000	1.0079	0
56	LIG	1	LIG	H19	HCMM	0.000000	1.0079	0
57	LIG	1	LIG	H20	HCMM	0.000000	1.0079	0
58	LIG	1	LIG	H21	HNCO	0.370000	1.0079	0
59	LIG	1	LIG	H22	HNCO	0.370000	1.0079	0
60	LIG	1	LIG	H23	HCMM	0.000000	1.0079	0
61	LIG	1	LIG	H24	HCMM	0.000000	1.0079	0
62	LIG	1	LIG	H25	HCMM	0.000000	1.0079	0
63	LIG	1	LIG	H26	HCMM	0.150000	1.0079	0
64	LIG	1	LIG	H27	HCMM	0.150000	1.0079	0
65	LIG	1	LIG	H28	HCMM	0.150000	1.0079	0
66	LIG	1	LIG	H29	HCMM	-0.000000	1.0079	0

BONDS

C5B	C=O	380.558	1.4310
C5B	C5A	512.256	1.3770
C5B	N5B	320.682	1.3690
C=O	NC=O	419.491	1.3690
C=O	O=C	931.963	1.2220
CR	C5A	322.481	1.4710
CR	CR	306.432	1.5080
CR	HCMM	342.991	1.0930
CR	NC=O	335.651	1.4360
C5A	N5B	599.191	1.3130
C5A	STHI	258.287	1.7170
C5A	HCMM	398.045	1.0800
HNCO	NC=O	479.511	1.0150

ANGLES

C5A	C5B	N5B	74.700	111.6210
C5A	C5B	C=O	59.588	124.8900
N5B	C5B	C=O	68.295	121.8210
N5B	C5A	STHI	61.459	114.5160

N5B C5A CR 62.250 127.6100
STHI C5A CR 64.913 122.1010
C5B C5A STHI 61.387 108.4800
C5B C5A HCMM 41.524 131.7210
STHI C5A HCMM 28.283 126.1410
C5B N5B C5A 86.791 103.7790
C5A STHI C5A 141.197 88.4950
C5B C=O NC=O 79.018 113.2330
C5B C=O O=C 77.075 124.1330
NC=O C=O O=C 65.273 127.1520
C=O NC=O CR 59.084 119.6000
C=O NC=O HNCO 41.380 120.2770
CR NC=O HNCO 39.725 120.0660
C5A CR NC=O 71.966 109.5000
C5A CR CR 72.397 110.0580
C5A CR HCMM 44.691 110.4670
NC=O CR CR 75.564 109.9600
NC=O CR HCMM 53.255 107.6460
CR CR HCMM 45.770 110.5490
CR CR CR 61.243 109.6080
HCMM CR HCMM 37.134 108.8360

DIHEDRALS
C5B C5A STHI C5A 3.500 2 180.00
C5B N5B C5A STHI 3.500 2 180.00
C5B N5B C5A CR 3.500 2 180.00
C5B C=O NC=O CR 3.000 2 180.00
C5B C=O NC=O HNCO 3.000 2 180.00
C5A N5B C5B C5A 3.500 2 180.00
C5A N5B C5B C=O 3.500 2 180.00
C5A STHI C5A HCMM 3.500 2 180.00
C5A CR NC=O C=O 0.500 3 0.00
C5A CR NC=O HNCO 0.150 3 0.00
C5A CR CR CR 0.150 3 0.00
C5A CR CR HCMM 0.150 3 0.00
C5A C5B C=O NC=O 1.250 2 180.00
C5A C5B C=O O=C 1.250 2 180.00
C5A STHI C5A N5B 3.500 2 180.00
C5A STHI C5A CR 3.500 2 180.00
N5B C5B C5A STHI 3.500 2 180.00
N5B C5B C5A HCMM 3.500 2 180.00
N5B C5B C=O NC=O 1.250 2 180.00
N5B C5B C=O O=C 1.250 2 180.00
N5B C5A CR NC=O 0.000 1 0.00
N5B C5A CR CR 0.000 1 0.00
N5B C5A CR HCMM 0.000 1 0.00
STHI C5A CR NC=O 0.000 1 0.00
STHI C5A CR CR 0.000 1 0.00

```
STHI C5A  CR   HCMM     0.000  1     0.00
STHI C5A  C5B  C=O      3.500  2   180.00
C=O  C5B  C5A  HCMM     0.000  1     0.00
C=O  NC=O CR   CR      -0.513  1     0.00
C=O  NC=O CR   CR       0.347  2   180.00
C=O  NC=O CR   CR       0.474  3     0.00
C=O  NC=O CR   HCMM    -1.050  1     0.00
C=O  NC=O CR   HCMM     0.681  2   180.00
C=O  NC=O CR   HCMM     0.011  3     0.00
NC=O CR   CR   CR       0.150  3     0.00
NC=O CR   CR   HCMM     0.213  3     0.00
CR   NC=O C=O  O=C     -0.160  1     0.00
CR   NC=O C=O  O=C      3.147  2   180.00
CR   NC=O C=O  O=C     -0.073  3     0.00
CR   CR   CR   HCMM     0.320  1     0.00
CR   CR   CR   HCMM    -0.315  2   180.00
CR   CR   CR   HCMM     0.132  3     0.00
O=C  C=O  NC=O HNCO     0.718  1     0.00
O=C  C=O  NC=O HNCO     2.487  2   180.00
O=C  C=O  NC=O HNCO    -0.227  3     0.00
CR   CR   NC=O HNCO     0.276  1     0.00
CR   CR   NC=O HNCO    -0.190  2   180.00
CR   CR   NC=O HNCO     0.163  3     0.00
HCMM CR   CR   HCMM     0.142  1     0.00
HCMM CR   CR   HCMM    -0.693  2   180.00
HCMM CR   CR   HCMM     0.157  3     0.00
HNCO NC=O CR   HCMM    -0.308  1     0.00
HNCO NC=O CR   HCMM     0.137  3     0.00
```

IMPROPER

```
C5B  N5B  C=O  C5A      2.879  0     0.00
C5A  CR   N5B  STHI     3.598  0     0.00
C=O  NC=O C5B  O=C      9.356  0     0.00
NC=O CR   C=O  HNCO    -1.439  0     0.00
CR   C5A  NC=O CR       0.000  0     0.00
CR   C5A  NC=O HCMM     0.000  0     0.00
C5A  N5B  CR   STHI     3.598  0     0.00
C5A  C5B  STHI HCMM     1.008  0     0.00
C5B  N5B  C5A  C=O      2.879  0     0.00
C=O  O=C  C5B  NC=O     9.356  0     0.00
CR   CR   CR   CR       0.000  0     0.00
CR   CR   CR   HCMM     0.000  0     0.00
CR   CR   C5A  HCMM     0.000  0     0.00
CR   HCMM CR   HCMM     0.000  0     0.00
C5A  STHI C5B  HCMM     1.008  0     0.00
```

NONBONDED nbxmod 5 atom cdiel shift vatom vdistance vswitch –
cutnb 14.0 ctofnb 12.0 ctonnb 10.0 eps 1.0 e14fac 1.0 wmin 1.5

```
C5B    0.000000  -0.050000    2.040000
C5A    0.000000  -0.050000    2.040000
N5B    0.000000  -0.200000    1.850000
STHI   0.000000  -0.450000    2.000000
C=O    0.000000  -0.110000    2.000000
NC=O   0.000000  -0.200000    1.850000
CR     0.000000  -0.055000    2.175000   0.000000  -0.010000    1.900000
O=C    0.000000  -0.120000    1.700000   0.000000  -0.120000    1.400000
HCMM   0.000000  -0.022000    1.320000
HNCO   0.000000  -0.046000    0.224500
```

3. Irinotecan

PSF file

```
    82 !NATOM
     1 LIG  1    LIG  C3   CB    -0.143500      12.0110         0
     2 LIG  1    LIG  C4   CB     0.000000      12.0110         0
     3 LIG  1    LIG  C5   CB    -0.150000      12.0110         0
     4 LIG  1    LIG  C6   CB     0.082500      12.0110         0
     5 LIG  1    LIG  C7   CB    -0.150000      12.0110         0
     6 LIG  1    LIG  C8   CB    -0.150000      12.0110         0
     7 LIG  1    LIG  C9   CB     0.310000      12.0110         0
     8 LIG  1    LIG  N1   NPYD  -0.620000      14.0067         0
     9 LIG  1    LIG  C10  CB     0.338400      12.0110         0
    10 LIG  1    LIG  C11  CB    -0.143500      12.0110         0
    11 LIG  1    LIG  N2   NC=O  -0.469100      14.0067         0
    12 LIG  1    LIG  C13  C=C    0.080600      12.0110         0
    13 LIG  1    LIG  C14  C=C   -0.150000      12.0110         0
    14 LIG  1    LIG  C15  C=C   -0.138200      12.0110         0
    15 LIG  1    LIG  C16  C=C   -0.123800      12.0110         0
    16 LIG  1    LIG  C17  C=O    0.615600      12.0110         0
    17 LIG  1    LIG  C1   CR     0.000000      12.0110         0
    18 LIG  1    LIG  C2   CR     0.143500      12.0110         0
    19 LIG  1    LIG  C12  CR     0.443600      12.0110         0
    20 LIG  1    LIG  O1   O=C   -0.570000      15.9994         0
    21 LIG  1    LIG  C18  CR     0.418200      12.0110         0
    22 LIG  1    LIG  O2   OR    -0.430000      15.9994         0
    23 LIG  1    LIG  C19  C=O    0.659000      12.0110         0
    24 LIG  1    LIG  O3   O=C   -0.570000      15.9994         0
    25 LIG  1    LIG  C20  CR     0.479200      12.0110         0
    26 LIG  1    LIG  C21  CR     0.000000      12.0110         0
    27 LIG  1    LIG  C22  CR     0.000000      12.0110         0
    28 LIG  1    LIG  O4   OR    -0.680000      15.9994         0
    29 LIG  1    LIG  O5   OR    -0.232500      15.9994         0
    30 LIG  1    LIG  C23  C=O    0.780000      12.0110         0
    31 LIG  1    LIG  O6   O=C   -0.570000      15.9994         0
    32 LIG  1    LIG  N3   NC=O  -0.660200      14.0067         0
    33 LIG  1    LIG  C24  CR     0.300100      12.0110         0
    34 LIG  1    LIG  C25  CR     0.000000      12.0110         0
    35 LIG  1    LIG  C26  CR     0.503000      12.0110         0
```

36	LIG	1	LIG	C27	CR	0.000000	12.0110	0
37	LIG	1	LIG	C28	CR	0.300100	12.0110	0
38	LIG	1	LIG	C29	CR	0.503000	12.0110	0
39	LIG	1	LIG	C30	CR	0.000000	12.0110	0
40	LIG	1	LIG	C31	CR	0.000000	12.0110	0
41	LIG	1	LIG	C32	CR	0.000000	12.0110	0
42	LIG	1	LIG	C33	CR	0.503000	12.0110	0
43	LIG	1	LIG	H1	HCMM	0.000000	1.0079	0
44	LIG	1	LIG	H2	HCMM	0.000000	1.0079	0
45	LIG	1	LIG	H3	HCMM	0.000000	1.0079	0
46	LIG	1	LIG	H4	HCMM	0.000000	1.0079	0
47	LIG	1	LIG	H5	HCMM	0.000000	1.0079	0
48	LIG	1	LIG	H6	HCMM	0.150000	1.0079	0
49	LIG	1	LIG	H7	HCMM	0.150000	1.0079	0
50	LIG	1	LIG	H8	HCMM	0.150000	1.0079	0
51	LIG	1	LIG	H9	HCMM	0.000000	1.0079	0
52	LIG	1	LIG	H10	HCMM	0.000000	1.0079	0
53	LIG	1	LIG	H11	HCMM	0.150000	1.0079	0
54	LIG	1	LIG	H12	HCMM	0.000000	1.0079	0
55	LIG	1	LIG	H13	HCMM	0.000000	1.0079	0
56	LIG	1	LIG	H14	HCMM	0.000000	1.0079	0
57	LIG	1	LIG	H15	HCMM	0.000000	1.0079	0
58	LIG	1	LIG	H16	HCMM	0.000000	1.0079	0
59	LIG	1	LIG	H17	HCMM	0.000000	1.0079	0
60	LIG	1	LIG	H18	HCMM	0.000000	1.0079	0
61	LIG	1	LIG	H19	HOR	0.400000	1.0079	0
62	LIG	1	LIG	H20	HCMM	0.000000	1.0079	0
63	LIG	1	LIG	H21	HCMM	0.000000	1.0079	0
64	LIG	1	LIG	H22	HCMM	0.000000	1.0079	0
65	LIG	1	LIG	H23	HCMM	0.000000	1.0079	0
66	LIG	1	LIG	H24	HCMM	0.000000	1.0079	0
67	LIG	1	LIG	H25	HCMM	0.000000	1.0079	0
68	LIG	1	LIG	H26	HCMM	0.000000	1.0079	0
69	LIG	1	LIG	H27	HCMM	0.000000	1.0079	0
70	LIG	1	LIG	H28	HCMM	0.000000	1.0079	0
71	LIG	1	LIG	H29	HCMM	0.000000	1.0079	0
72	LIG	1	LIG	H30	HCMM	0.000000	1.0079	0
73	LIG	1	LIG	H31	HCMM	0.000000	1.0079	0
74	LIG	1	LIG	H32	HCMM	0.000000	1.0079	0
75	LIG	1	LIG	H33	HCMM	0.000000	1.0079	0
76	LIG	1	LIG	H34	HCMM	0.000000	1.0079	0
77	LIG	1	LIG	H35	HCMM	0.000000	1.0079	0
78	LIG	1	LIG	H36	HCMM	0.000000	1.0079	0
79	LIG	1	LIG	H37	HCMM	0.000000	1.0079	0
80	LIG	1	LIG	H38	HCMM	0.000000	1.0079	0
81	LIG	1	LIG	N4	NRP	-0.959000	14.0067	0
82	LIG	1	LIG	H39	HNRP	0.450000	1.0079	0

BONDS

CR CR 306.432 1.5080
CR HCMM 342.991 1.0930
CR CB 356.737 1.4860
CB CB 401.068 1.3740
CB HCMM 381.853 1.0840
CB OR 404.019 1.3760
CB NPYD 412.870 1.3330
CB C=C 360.335 1.4490
CR NC=O 335.651 1.4360
NC=O C=O 419.491 1.3690
NC=O C=C 455.474 1.3620
C=C C=C 684.039 1.3330
C=C HCMM 372.066 1.0830
C=C CR 326.655 1.4820
C=C C=O 328.526 1.4680
C=O O=C 931.963 1.2220
CR OR 363.214 1.4180
OR C=O 417.476 1.3550
C=O CR 301.539 1.4920
OR HOR 560.905 0.9720
CR NRP 276.638 1.4800
NRP HNRP 443.528 1.0280

ANGLES
CB CB CB 48.145 119.9770
CB CB CR 57.788 120.4190
CB CB HCMM 40.517 120.5710
CB CB OR 69.663 116.4950
CB CB NPYD 42.892 126.1390
CB NPYD CB 78.083 115.4060
NPYD CB C=C 74.053 117.2200
CB CB C=C 51.240 119.6950
C=C NC=O C=O 71.966 120.7030
C=C NC=O CR 72.254 118.9160
C=O NC=O CR 59.084 119.6000
CB C=C NC=O 73.477 117.1390
CB C=C C=C 43.035 117.5080
NC=O C=C C=C 72.182 120.8280
C=C C=C C=C 53.758 121.5500
C=C C=C HCMM 38.502 121.0040
C=C C=C CR 49.225 116.9290
C=C C=C C=O 39.221 111.2970
C=O C=C CR 50.232 116.1040
NC=O C=O C=C 74.988 111.7210
NC=O C=O O=C 65.273 127.1520
C=C C=O O=C 67.360 122.6230
CR CR HCMM 45.770 110.5490
HCMM CR HCMM 37.134 108.8360

CB CR CR 54.406 108.6170
CB CR HCMM 45.122 109.4910
CB CR NC=O 79.666 110.4230
NC=O CR HCMM 53.255 107.6460
C=C CR OR 77.291 108.6990
C=C CR HCMM 45.482 110.2920
OR CR HCMM 56.205 108.5770
CR OR C=O 66.424 108.0550
OR C=O O=C 83.120 124.4250
OR C=O CR 75.060 109.7160
O=C C=O CR 67.504 124.4100
C=C CR C=O 48.001 104.8290
C=C CR CR 52.967 109.4450
C=O CR CR 55.917 107.5170
C=O CR OR 37.998 104.1120
CR CR OR 71.390 108.1330
CR CR CR 61.243 109.6080
CR OR HOR 57.069 106.5030
CB OR C=O 44.187 95.3000
OR C=O NC=O 101.112 112.1870
CR NC=O CR 80.386 117.9090
NC=O CR CR 75.564 109.9600
CR CR NRP 84.848 106.4930
HCMM CR NRP 62.754 106.2240
CR NRP CR 62.034 112.2510
CR NRP HNRP 41.452 111.2060

DIHEDRALS
CB CB CB CB 3.500 2 180.00
CB CB CB HCMM 3.500 2 180.00
CB CB CB NPYD 3.500 2 180.00
CB CB CB C=C 3.500 2 180.00
CB CB CR NC=O 0.100 3 0.00
CB CB CR HCMM -0.210 2 180.00
CB CB CR HCMM 0.196 3 0.00
CB CR CR HCMM 0.195 3 0.00
CB CB CB CR 3.500 2 180.00
CB CB CR CR 0.225 2 180.00
CB CB CB OR 3.500 2 180.00
CB CB NPYD CB 3.500 2 180.00
CB CB OR C=O 1.288 2 180.00
CB OR C=O O=C 0.318 1 0.00
CB OR C=O O=C 2.945 2 180.00
CB OR C=O O=C -0.223 3 0.00
CB OR C=O NC=O 2.750 2 180.00
CB NPYD CB C=C 3.500 2 180.00
NPYD CB CB HCMM 3.500 2 180.00
NPYD CB CB CR 3.500 2 180.00

```
NPYD CB   C=C  NC=O     1.000  2   180.00
NPYD CB   C=C  C=C      1.000  2   180.00
CB   C=C  NC=O C=O      3.000  2   180.00
CB   C=C  NC=O CR       3.000  2   180.00
CB   C=C  C=C  C=C      0.900  2   180.00
CB   C=C  C=C  HCMM     0.900  2   180.00
CB   CB   C=C  NC=O     1.000  2   180.00
CB   CB   C=C  C=C      0.771  2   180.00
CB   CB   C=C  C=C      0.217  3     0.00
CB   CR   NC=O C=C      0.150  3     0.00
CB   CR   NC=O C=O      0.500  3     0.00
NC=O C=C  C=C  C=C      6.000  2   180.00
NC=O C=C  C=C  HCMM     6.000  2   180.00
NC=O C=O  C=C  C=C      1.250  2   180.00
NC=O C=O  C=C  CR       1.250  2   180.00
C=C  CB   CB   CR       1.000  2   180.00
C=C  NC=O C=O  C=C      3.000  2   180.00
C=C  NC=O C=O  O=C      3.000  2   180.00
C=C  NC=O CR   HCMM     0.150  3     0.00
C=C  C=C  C=C  C=C      0.047  1     0.00
C=C  C=C  C=C  C=C      0.810  2   180.00
C=C  C=C  C=C  C=C      0.439  3     0.00
C=C  C=C  C=C  CR      -0.209  1     0.00
C=C  C=C  C=C  CR       1.044  2   180.00
C=C  C=C  C=C  CR      -0.155  3     0.00
C=C  C=C  NC=O C=O      3.000  2   180.00
C=C  C=C  NC=O CR       3.000  2   180.00
C=C  C=C  C=C  C=O      0.900  2   180.00
C=C  C=C  CR   C=O      0.000  1     0.00
C=C  C=C  CR   CR       0.000  1     0.00
C=C  C=C  CR   OR       0.000  1     0.00
C=C  C=C  C=O  O=C      0.181  1     0.00
C=C  C=C  C=O  O=C      0.989  2   180.00
C=C  C=C  CR   HCMM     0.251  1     0.00
C=C  C=C  CR   HCMM    -0.205  2   180.00
C=C  C=C  CR   HCMM    -0.268  3     0.00
C=C  CR   C=O  OR       0.200  2   180.00
C=C  CR   C=O  OR       0.150  3     0.00
C=C  CR   C=O  O=C     -0.379  1     0.00
C=C  CR   C=O  O=C      0.056  2   180.00
C=C  CR   C=O  O=C      0.281  3     0.00
C=C  CR   CR   CR      -0.147  1     0.00
C=C  CR   CR   CR       0.219  2   180.00
C=C  CR   CR   CR       0.292  3     0.00
C=C  CR   CR   HCMM     0.161  1     0.00
C=C  CR   CR   HCMM    -0.205  2   180.00
C=C  CR   CR   HCMM     0.072  3     0.00
C=C  CR   OR   HOR      0.051  1     0.00
```

```
C=C  CR   OR   HOR      0.230  2   180.00
C=C  CR   OR   HOR     -0.064  3     0.00
C=C  C=C  C=C  HCMM     6.000  2   180.00
C=C  C=O  NC=O CR       3.000  2   180.00
C=C  CR   OR   C=O      0.100  3     0.00
C=O  NC=O CR   HCMM    -1.050  1     0.00
C=O  NC=O CR   HCMM     0.681  2   180.00
C=O  NC=O CR   HCMM     0.011  3     0.00
C=O  C=C  C=C  CR       0.900  2   180.00
C=O  C=C  CR   OR       0.000  1     0.00
C=O  C=C  CR   HCMM    -0.054  3     0.00
CR   CB   CB   CR       3.500  2   180.00
CR   NC=O C=O  O=C     -0.160  1     0.00
CR   NC=O C=O  O=C      3.147  2   180.00
CR   NC=O C=O  O=C     -0.073  3     0.00
O=C  C=O  C=C  CR       1.250  2   180.00
CR   C=C  C=C  CR      -0.202  1     0.00
CR   C=C  C=C  CR       6.000  2   180.00
CR   OR   C=O  O=C      0.341  1     0.00
CR   OR   C=O  O=C      3.592  2   180.00
CR   OR   C=O  O=C     -0.468  3     0.00
CR   OR   C=O  CR      -0.622  1     0.00
CR   OR   C=O  CR       2.741  2   180.00
CR   OR   C=O  CR       0.182  3     0.00
OR   C=O  CR   CR      -0.059  1     0.00
OR   C=O  CR   CR      -0.167  2   180.00
OR   C=O  CR   CR       0.101  3     0.00
OR   C=O  CR   OR       0.224  1     0.00
OR   C=O  CR   OR       0.326  2   180.00
OR   C=O  CR   OR       0.159  3     0.00
C=O  OR   CR   HCMM     0.286  1     0.00
C=O  OR   CR   HCMM    -0.152  3     0.00
C=O  CR   CR   CR       0.033  1     0.00
C=O  CR   CR   CR      -0.078  2   180.00
C=O  CR   CR   CR       0.071  3     0.00
C=O  CR   CR   HCMM    -0.128  1     0.00
C=O  CR   CR   HCMM     0.029  2   180.00
C=O  CR   OR   HOR     -0.826  1     0.00
C=O  CR   OR   HOR     -0.830  2   180.00
C=O  CR   OR   HOR      0.141  3     0.00
O=C  C=O  CR   CR       0.412  1     0.00
O=C  C=O  CR   CR       0.070  2   180.00
O=C  C=O  CR   CR       0.163  3     0.00
O=C  C=O  CR   OR      -0.198  1     0.00
O=C  C=O  CR   OR       0.365  2   180.00
O=C  C=O  CR   OR      -0.070  3     0.00
CR   C=C  C=C  HCMM     6.000  2   180.00
CR   CR   CR   HCMM     0.320  1     0.00
```

```
CR   CR   CR   HCMM    -0.315  2   180.00
CR   CR   CR   HCMM     0.132  3     0.00
CR   CR   OR   HOR      0.135  2   180.00
CR   CR   OR   HOR      0.118  3     0.00
CR   CR   CR   OR      -0.344  1     0.00
CR   CR   CR   OR       0.879  2   180.00
CR   CR   CR   OR       0.238  3     0.00
OR   CR   CR   HCMM    -0.327  1     0.00
OR   CR   CR   HCMM     0.536  2   180.00
OR   CR   CR   HCMM     0.140  3     0.00
OR   CB   CB   HCMM     3.500  2   180.00
OR   C=O  NC=O CR       3.000  2   180.00
C=O  NC=O CR   CR      -0.513  1     0.00
C=O  NC=O CR   CR       0.347  2   180.00
C=O  NC=O CR   CR       0.474  3     0.00
NC=O CR   CR   CR       0.150  3     0.00
NC=O CR   CR   HCMM     0.213  3     0.00
CR   NC=O CR   CR       0.150  3     0.00
CR   NC=O CR   HCMM     0.390  3     0.00
CR   CR   CR   CR       0.051  1     0.00
CR   CR   CR   CR       0.341  2   180.00
CR   CR   CR   CR       0.166  3     0.00
CR   CR   CR   NRP     -0.324  1     0.00
CR   CR   CR   NRP      0.275  2   180.00
CR   CR   CR   NRP      0.295  3     0.00
CR   CR   NRP  CR       0.125  3     0.00
CR   CR   NRP  HNRP     0.093  3     0.00
CR   NRP  CR   HCMM     0.123  3     0.00
HCMM CR   CR   HCMM     0.142  1     0.00
HCMM CR   CR   HCMM    -0.693  2   180.00
HCMM CR   CR   HCMM     0.157  3     0.00
HCMM CB   CB   HCMM     3.500  2   180.00
HCMM CR   CR   NRP      0.346  1     0.00
HCMM CR   CR   NRP     -0.265  2   180.00
HCMM CR   CR   NRP      0.139  3     0.00
HCMM CR   NRP  HNRP     0.130  3     0.00
```

IMPROPER

```
CB   CB   CB   CR       2.879  0     0.00
CB   CB   CB   CB       2.519  0     0.00
CB   CB   CB   HCMM     1.079  0     0.00
CB   OR   CB   CB       3.454  0     0.00
CB   CB   CB   NPYD     2.519  0     0.00
CB   CB   NPYD C=C      2.519  0     0.00
C=C  C=C  CB   NC=O     1.439  0     0.00
C=C  C=C  C=C  HCMM     0.936  0     0.00
C=C  CR   C=C  C=C      1.943  0     0.00
NC=O C=O  C=C  CR      -1.439  0     0.00
```

```
CR   CR   CB   HCMM     0.000  0     0.00
C=O  C=C  NC=O O=C      8.348  0     0.00
C=C  C=O  C=C  CR       1.871  0     0.00
CR   OR   C=C  HCMM     0.000  0     0.00
C=O  CR   OR   O=C     10.147  0     0.00
CR   C=O  C=C  CR       0.000  0     0.00
CR   C=O  C=C  OR       0.000  0     0.00
CR   CR   CR   HCMM     0.000  0     0.00
C=O  NC=O OR   O=C      9.356  0     0.00
NC=O CR   C=O  CR      -1.439  0     0.00
CR   CR   NC=O HCMM     0.000  0     0.00
CR   NRP  CR   CR       0.000  0     0.00
NRP  CR   CR   CR       0.000  0     0.00
NRP  CR   CR   HNRP     0.000  0     0.00
CR   CR   NRP  HCMM     0.000  0     0.00
CR   HCMM CR   HCMM     0.000  0     0.00
CR   CB   NC=O HCMM     0.000  0     0.00
```

NONBONDED nbxmod 5 atom cdiel shift vatom vdistance vswitch -
cutnb 14.0 ctofnb 12.0 ctonnb 10.0 eps 1.0 e14fac 1.0 wmin 1.5

```
CB      0.000000  -0.070000     1.992400
NPYD    0.000000  -0.200000     1.850000
NC=O    0.000000  -0.200000     1.850000
C=C     0.000000  -0.068000     2.090000
C=O     0.000000  -0.110000     2.000000
CR      0.000000  -0.055000     2.175000   0.000000  -0.010000     1.900000
O=C     0.000000  -0.120000     1.700000   0.000000  -0.120000     1.400000
OR      0.000000  -0.152100     1.770000
HCMM    0.000000  -0.022000     1.320000
HOR     0.000000  -0.046000     0.224500
NRP     0.000000  -0.200000     1.850000
HNRP    0.000000  -0.046000     0.224500
```

4. Vinblastine

PSF file

```
     118 !NATOM
       1 LIG  1    LIG  C8   C5A   -0.331600      12.0110         0
       2 LIG  1    LIG  C9   C5B   -0.181000      12.0110         0
       3 LIG  1    LIG  C10  C5B    0.000000      12.0110         0
       4 LIG  1    LIG  C11  CB    -0.150000      12.0110         0
       5 LIG  1    LIG  C12  CB    -0.150000      12.0110         0
       6 LIG  1    LIG  C13  CB    -0.150000      12.0110         0
       7 LIG  1    LIG  C14  CB    -0.150000      12.0110         0
       8 LIG  1    LIG  C15  C5A   -0.151600      12.0110         0
       9 LIG  1    LIG  N1   NPYL   0.033200      14.0067         0
      10 LIG  1    LIG  C20  CB    -0.143500      12.0110         0
      11 LIG  1    LIG  C21  CB    -0.150000      12.0110         0
      12 LIG  1    LIG  C22  CB    -0.143500      12.0110         0
      13 LIG  1    LIG  C23  CB     0.100000      12.0110         0
```

14	LIG	1	LIG	C24	CB	-0.150000	12.0110	0
15	LIG	1	LIG	C25	CB	0.082500	12.0110	0
16	LIG	1	LIG	C1	CR	0.000000	12.0110	0
17	LIG	1	LIG	C2	CR	0.000000	12.0110	0
18	LIG	1	LIG	C3	CR	0.280000	12.0110	0
19	LIG	1	LIG	C4	CR	0.000000	12.0110	0
20	LIG	1	LIG	C5	CR	0.000000	12.0110	0
21	LIG	1	LIG	H1	HCMM	0.000000	1.0079	0
22	LIG	1	LIG	C6	CR	0.000000	12.0110	0
23	LIG	1	LIG	C7	CR	0.384500	12.0110	0
24	LIG	1	LIG	H2	HNR	0.270000	1.0079	0
25	LIG	1	LIG	C16	CR	0.181000	12.0110	0
26	LIG	1	LIG	C17	CR	0.503000	12.0110	0
27	LIG	1	LIG	N2	NRP	-0.959000	14.0067	0
28	LIG	1	LIG	C18	CR	0.503000	12.0110	0
29	LIG	1	LIG	C19	CR	0.503000	12.0110	0
30	LIG	1	LIG	O1	OR	-0.362500	15.9994	0
31	LIG	1	LIG	C26	CR	0.280000	12.0110	0
32	LIG	1	LIG	N3	NC=C	-0.838200	14.0067	0
33	LIG	1	LIG	C27	CR	0.369100	12.0110	0
34	LIG	1	LIG	H3	HCMM	0.000000	1.0079	0
35	LIG	1	LIG	C28	CR	0.143500	12.0110	0
36	LIG	1	LIG	C29	CR	0.000000	12.0110	0
37	LIG	1	LIG	C30	CR	0.270000	12.0110	0
38	LIG	1	LIG	N4	NR	-0.810000	14.0067	0
39	LIG	1	LIG	C31	CR	0.270000	12.0110	0
40	LIG	1	LIG	H4	HCMM	0.000000	1.0079	0
41	LIG	1	LIG	C32	CR	0.138200	12.0110	0
42	LIG	1	LIG	C33	C=C	-0.288200	12.0110	0
43	LIG	1	LIG	C34	C=C	-0.288200	12.0110	0
44	LIG	1	LIG	C35	CR	0.408200	12.0110	0
45	LIG	1	LIG	C36	CR	0.280000	12.0110	0
46	LIG	1	LIG	H5	HCMM	0.000000	1.0079	0
47	LIG	1	LIG	C37	CR	0.341000	12.0110	0
48	LIG	1	LIG	C38	C=O	0.659000	12.0110	0
49	LIG	1	LIG	O2	O=C	-0.570000	15.9994	0
50	LIG	1	LIG	O3	OR	-0.430000	15.9994	0
51	LIG	1	LIG	C39	CR	0.280000	12.0110	0
52	LIG	1	LIG	O4	OR	-0.680000	15.9994	0
53	LIG	1	LIG	O5	OR	-0.430000	15.9994	0
54	LIG	1	LIG	C40	C=O	0.659000	12.0110	0
55	LIG	1	LIG	O6	O=C	-0.570000	15.9994	0
56	LIG	1	LIG	C41	CR	0.061000	12.0110	0
57	LIG	1	LIG	C42	CR	0.000000	12.0110	0
58	LIG	1	LIG	C43	CR	0.000000	12.0110	0
59	LIG	1	LIG	C44	CR	0.369100	12.0110	0
60	LIG	1	LIG	C45	C=O	0.659000	12.0110	0
61	LIG	1	LIG	O7	O=C	-0.570000	15.9994	0

62	LIG	1	LIG	O8	OR	-0.430000	15.9994	0
63	LIG	1	LIG	C46	CR	0.280000	12.0110	0
64	LIG	1	LIG	O9	OR	-0.680000	15.9994	0
65	LIG	1	LIG	H6	HCMM	0.000000	1.0079	0
66	LIG	1	LIG	H7	HCMM	0.000000	1.0079	0
67	LIG	1	LIG	H8	HCMM	0.000000	1.0079	0
68	LIG	1	LIG	H9	HCMM	0.000000	1.0079	0
69	LIG	1	LIG	H10	HCMM	0.000000	1.0079	0
70	LIG	1	LIG	H11	HCMM	0.000000	1.0079	0
71	LIG	1	LIG	H12	HCMM	0.000000	1.0079	0
72	LIG	1	LIG	H13	HCMM	0.000000	1.0079	0
73	LIG	1	LIG	H14	HCMM	0.000000	1.0079	0
74	LIG	1	LIG	H15	HCMM	0.150000	1.0079	0
75	LIG	1	LIG	H16	HCMM	0.150000	1.0079	0
76	LIG	1	LIG	H17	HCMM	0.150000	1.0079	0
77	LIG	1	LIG	H18	HCMM	0.150000	1.0079	0
78	LIG	1	LIG	H19	HCMM	0.000000	1.0079	0
79	LIG	1	LIG	H20	HCMM	0.000000	1.0079	0
80	LIG	1	LIG	H21	HCMM	0.000000	1.0079	0
81	LIG	1	LIG	H22	HCMM	0.000000	1.0079	0
82	LIG	1	LIG	H23	HCMM	0.000000	1.0079	0
83	LIG	1	LIG	H24	HCMM	0.000000	1.0079	0
84	LIG	1	LIG	H25	HCMM	0.000000	1.0079	0
85	LIG	1	LIG	H26	HCMM	0.000000	1.0079	0
86	LIG	1	LIG	H27	HCMM	0.150000	1.0079	0
87	LIG	1	LIG	H28	HCMM	0.150000	1.0079	0
88	LIG	1	LIG	H29	HCMM	0.000000	1.0079	0
89	LIG	1	LIG	H30	HCMM	0.000000	1.0079	0
90	LIG	1	LIG	H31	HCMM	0.000000	1.0079	0
91	LIG	1	LIG	H32	HCMM	0.000000	1.0079	0
92	LIG	1	LIG	H33	HCMM	0.000000	1.0079	0
93	LIG	1	LIG	H34	HCMM	0.000000	1.0079	0
94	LIG	1	LIG	H35	HCMM	0.000000	1.0079	0
95	LIG	1	LIG	H36	HCMM	0.150000	1.0079	0
96	LIG	1	LIG	H37	HCMM	0.150000	1.0079	0
97	LIG	1	LIG	H38	HCMM	0.000000	1.0079	0
98	LIG	1	LIG	H39	HCMM	0.000000	1.0079	0
99	LIG	1	LIG	H40	HCMM	0.000000	1.0079	0
100	LIG	1	LIG	H41	HCMM	0.000000	1.0079	0
101	LIG	1	LIG	H42	HCMM	0.000000	1.0079	0
102	LIG	1	LIG	H43	HOR	0.400000	1.0079	0
103	LIG	1	LIG	H44	HCMM	0.000000	1.0079	0
104	LIG	1	LIG	H45	HCMM	0.000000	1.0079	0
105	LIG	1	LIG	H46	HCMM	0.000000	1.0079	0
106	LIG	1	LIG	H47	HCMM	0.000000	1.0079	0
107	LIG	1	LIG	H48	HCMM	0.000000	1.0079	0
108	LIG	1	LIG	H49	HCMM	0.000000	1.0079	0
109	LIG	1	LIG	H50	HCMM	0.000000	1.0079	0

110 LIG 1 LIG H51 HCMM 0.000000 1.0079 0
111 LIG 1 LIG H52 HCMM 0.000000 1.0079 0
112 LIG 1 LIG H53 HCMM 0.000000 1.0079 0
113 LIG 1 LIG H54 HCMM 0.000000 1.0079 0
114 LIG 1 LIG H55 HCMM 0.000000 1.0079 0
115 LIG 1 LIG H56 HCMM 0.000000 1.0079 0
116 LIG 1 LIG H57 HCMM 0.000000 1.0079 0
117 LIG 1 LIG H58 HOR 0.400000 1.0079 0
118 LIG 1 LIG H59 HNRP 0.450000 1.0079 0

BONDS

CR CR 306.432 1.5080
CR HCMM 342.991 1.0930
CR OR 363.214 1.4180
CR C5A 322.481 1.4710
CR CB 356.737 1.4860
CR C=O 301.539 1.4920
C5A NPYL 453.459 1.3640
C5A C5B 512.256 1.3770
C5B C5B 310.390 1.4180
C5B CR 325.144 1.4690
C5B CB 443.384 1.3790
CB CB 401.068 1.3740
CB HCMM 381.853 1.0840
CB C5A 438.634 1.3720
NPYL HNR 511.824 1.0120
CR NRP 276.638 1.4800
NRP HNRP 443.528 1.0280
CB NC=C 443.888 1.3980
CB OR 404.019 1.3760
NC=C CR 354.218 1.4460
CR NR 365.876 1.4510
CR C=C 326.655 1.4820
C=C C=C 684.039 1.3330
C=C HCMM 372.066 1.0830
C=O O=C 931.963 1.2220
C=O OR 417.476 1.3550
OR HOR 560.905 0.9720

ANGLES

C5B C5A NPYL 58.508 107.2550
C5B C5A CR 53.039 131.3780
NPYL C5A CR 67.288 121.8320
C5A C5B C5B 62.322 108.2390
C5A C5B CR 55.845 128.0410
C5B C5B CR 55.126 128.0610
C5B C5B CB 61.459 136.0870
CB C5B C5A 65.201 117.9660
C5B CB CB 30.441 112.5670

C5B	CB	HCMM	37.638	121.4460
CB	CB	HCMM	40.517	120.5710
CB	CB	CB	48.145	119.9770
CB	CB	C5A	34.400	111.2430
C5A	CB	HCMM	50.520	121.2380
C5B	C5A	CB	48.865	122.8810
CB	C5A	NPYL	72.757	132.0460
C5A	NPYL	C5A	82.904	109.5990
C5A	NPYL	HNR	39.653	127.7700
CB	CB	CR	57.788	120.4190
CB	CB	NC=C	75.204	121.6330
CB	CB	OR	69.663	116.4950
CR	CR	HCMM	45.770	110.5490
HCMM	CR	HCMM	37.134	108.8360
CR	CR	CR	61.243	109.6080
CR	CR	OR	71.390	108.1330
C5A	CR	CB	71.966	109.5000
C5A	CR	CR	72.397	110.0580
C5A	CR	C=O	76.931	107.0770
CB	CR	CR	54.406	108.6170
CB	CR	C=O	72.757	109.8330
CR	CR	C=O	55.917	107.5170
C5B	CR	CR	71.102	111.0640
C5B	CR	HCMM	44.763	110.4570
CR	CR	NRP	84.848	106.4930
NRP	CR	HCMM	62.754	106.2240
CR	NRP	CR	62.034	112.2510
CR	NRP	HNRP	41.452	111.2060
CB	OR	CR	77.363	102.8460
OR	CR	HCMM	56.205	108.5770
CB	NC=C	CR	60.091	107.3490
CR	NC=C	CR	76.571	113.7030
NC=C	CR	HCMM	51.743	109.8700
NC=C	CR	CR	81.321	108.6780
CR	CR	NR	55.917	108.2900
NR	CR	HCMM	46.994	110.2970
CR	NR	CR	78.443	107.0180
CR	CR	C=C	52.967	109.4450
CR	C=C	C=C	48.361	122.1410
CR	C=C	HCMM	32.097	120.1080
C=C	C=C	HCMM	38.502	121.0040
NR	CR	C=C	63.618	111.5530
C=C	CR	HCMM	45.482	110.2920
C=O	CR	OR	37.998	104.1120
CR	C=O	O=C	67.504	124.4100
CR	C=O	OR	75.060	109.7160
O=C	C=O	OR	83.120	124.4250
C=O	OR	CR	66.424	108.0550

CR OR HOR 57.069 106.5030
C=O CR HCMM 46.778 108.3850

DIHEDRALS
C5A C5B C5B CB 3.500 2 180.00
C5A C5B C5B C5A 3.500 2 180.00
C5A C5B CR CR 0.000 1 0.00
C5A C5B CR HCMM 0.000 1 0.00
C5A NPYL C5A C5B 2.000 2 180.00
C5A NPYL C5A CB 2.000 2 180.00
C5A CR CB CB 0.100 3 0.00
C5A CR CR CR 0.150 3 0.00
C5A CR CR HCMM 0.150 3 0.00
C5A CR C=O O=C 0.200 2 180.00
C5A CR C=O O=C 0.200 3 0.00
C5A CR C=O OR 0.200 2 180.00
C5A CR C=O OR 0.150 3 0.00
C5B C5A NPYL HNR 2.000 2 180.00
C5B C5A CR CB 0.000 1 0.00
C5B C5A CR CR 0.000 1 0.00
C5B C5A CR C=O 0.000 1 0.00
C5B C5B CB CB 3.500 2 180.00
C5B C5B CB HCMM 3.500 2 180.00
C5B C5B C5A CB 3.500 2 180.00
C5B C5B C5A NPYL 3.500 2 180.00
C5B CR CR NRP 0.150 3 0.00
C5B CR CR HCMM 0.150 3 0.00
C5B C5B C5A CR 3.500 2 180.00
C5B C5B CR CR 0.000 1 0.00
C5B C5B CR HCMM 0.000 1 0.00
C5B CB CB CB 3.500 2 180.00
C5B CB CB HCMM 3.500 2 180.00
C5B C5A CB CB 3.500 2 180.00
C5B C5A CB HCMM 3.500 2 180.00
CB C5B C5B CR 3.500 2 180.00
CB C5B C5A CB 3.500 2 180.00
CB C5B C5A NPYL 3.500 2 180.00
CB CB CB CB 3.500 2 180.00
CB CB CB HCMM 3.500 2 180.00
CB CB C5B C5A 3.500 2 180.00
CB CB CB C5A 3.500 2 180.00
CB CB C5A NPYL 3.500 2 180.00
CB C5A NPYL HNR 2.000 2 180.00
C5A C5B C5B CR 3.500 2 180.00
C5A C5B CB HCMM 3.500 2 180.00
C5A CB CB HCMM 3.500 2 180.00
C5A NPYL C5A CR 2.000 2 180.00
NPYL C5A C5B CR 3.500 2 180.00

```
NPYL C5A  CR   CB       0.000  1     0.00
NPYL C5A  CR   CR       0.000  1     0.00
NPYL C5A  CR   C=O      0.000  1     0.00
NPYL C5A  CB   HCMM     3.500  2   180.00
CB   CB   CB   CR       3.500  2   180.00
CB   CB   OR   CR       2.191  2   180.00
CB   CR   CR   CR       0.150  3     0.00
CB   CR   CR   HCMM     0.195  3     0.00
CB   CR   C=O  O=C      0.200  2   180.00
CB   CR   C=O  O=C      0.200  3     0.00
CB   CR   C=O  OR       0.200  2   180.00
CB   CR   C=O  OR       0.150  3     0.00
CB   CB   CB   OR       3.500  2   180.00
CB   CB   CR   CR       0.225  2   180.00
CB   CB   CR   C=O      0.100  3     0.00
CB   CB   CB   NC=C     3.500  2   180.00
CB   CB   NC=C CR       2.168  2   180.00
CB   CB   NC=C CR       0.185  3     0.00
CB   CR   CR   NC=C     0.150  3     0.00
CB   CR   CR   NR       0.150  3     0.00
CB   NC=C CR   HCMM     0.165  3     0.00
CB   NC=C CR   CR       0.125  3     0.00
CB   OR   CR   HCMM     0.053  3     0.00
CR   CR   CR   CR       0.051  1     0.00
CR   CR   CR   CR       0.341  2   180.00
CR   CR   CR   CR       0.166  3     0.00
CR   CR   CR   OR      -0.344  1     0.00
CR   CR   CR   OR       0.879  2   180.00
CR   CR   CR   OR       0.238  3     0.00
CR   CR   CR   HCMM     0.320  1     0.00
CR   CR   CR   HCMM    -0.315  2   180.00
CR   CR   CR   HCMM     0.132  3     0.00
CR   CR   CR   NRP     -0.324  1     0.00
CR   CR   CR   NRP      0.275  2   180.00
CR   CR   CR   NRP      0.295  3     0.00
CR   CR   OR   HOR      0.135  2   180.00
CR   CR   OR   HOR      0.118  3     0.00
CR   CR   NRP  CR       0.125  3     0.00
CR   CR   NRP  HNRP     0.093  3     0.00
CR   CR   CR   C=O      0.033  1     0.00
CR   CR   CR   C=O     -0.078  2   180.00
CR   CR   CR   C=O      0.071  3     0.00
HCMM CR   CR   HCMM     0.142  1     0.00
HCMM CR   CR   HCMM    -0.693  2   180.00
HCMM CR   CR   HCMM     0.157  3     0.00
HCMM CR   CR   NRP      0.346  1     0.00
HCMM CR   CR   NRP     -0.265  2   180.00
HCMM CR   CR   NRP      0.139  3     0.00
```

```
CR   CR   C=O  O=C      0.412  1     0.00
CR   CR   C=O  O=C      0.070  2   180.00
CR   CR   C=O  O=C      0.163  3     0.00
CR   CR   C=O  OR      -0.059  1     0.00
CR   CR   C=O  OR      -0.167  2   180.00
CR   CR   C=O  OR       0.101  3     0.00
CR   C5A  C5B  CR       3.500  2   180.00
CR   C5A  NPYL HNR      2.000  2   180.00
CR   CB   CB   HCMM     3.500  2   180.00
CR   CB   CB   OR       3.500  2   180.00
CR   C=O  OR   CR      -0.622  1     0.00
CR   C=O  OR   CR       2.741  2   180.00
CR   C=O  OR   CR       0.182  3     0.00
CR   NRP  CR   HCMM     0.123  3     0.00
NRP  CR   CR   OR       0.150  3     0.00
OR   CB   CB   HCMM     3.500  2   180.00
NC=C CB   CB   CR       3.500  2   180.00
NC=C CB   CB   HCMM     3.500  2   180.00
NC=C CR   CR   CR       0.150  3     0.00
NC=C CR   CR   C=O      0.150  3     0.00
NC=C CR   CR   OR       0.150  3     0.00
CR   NC=C CR   HCMM     0.125  3     0.00
CR   CR   CR   NR      -0.710  1     0.00
CR   CR   CR   NR      -0.046  2   180.00
CR   CR   CR   NR       0.550  3     0.00
HCMM CR   CR   C=O     -0.128  1     0.00
HCMM CR   CR   C=O      0.029  2   180.00
HCMM CR   CR   OR      -0.327  1     0.00
HCMM CR   CR   OR       0.536  2   180.00
HCMM CR   CR   OR       0.140  3     0.00
CR   CR   NC=C CR       0.125  3     0.00
CR   CR   NR   CR      -0.220  1     0.00
CR   CR   NR   CR       0.393  2   180.00
CR   CR   NR   CR       0.136  3     0.00
CR   CR   CR   C=C     -0.147  1     0.00
CR   CR   CR   C=C      0.219  2   180.00
CR   CR   CR   C=C      0.292  3     0.00
CR   NR   CR   HCMM     0.197  1     0.00
CR   NR   CR   HCMM    -0.193  2   180.00
CR   NR   CR   HCMM     0.281  3     0.00
CR   NR   CR   C=C     -0.150  2   180.00
CR   NR   CR   C=C      0.250  3     0.00
NR   CR   CR   HCMM    -0.372  1     0.00
NR   CR   CR   HCMM    -0.617  2   180.00
NR   CR   CR   HCMM     0.169  3     0.00
NR   CR   CR   C=C      0.150  3     0.00
NR   CR   C=C  C=C      0.271  1     0.00
NR   CR   C=C  C=C      0.270  2   180.00
```

NR	CR	C=C	C=C	-0.504	3	0.00
NR	CR	C=C	HCMM	0.102	2	180.00
NR	CR	C=C	HCMM	0.232	3	0.00
CR	CR	C=C	C=C	-0.247	1	0.00
CR	CR	C=C	C=C	0.137	2	180.00
CR	CR	C=C	C=C	-0.315	3	0.00
CR	CR	C=C	HCMM	0.037	1	0.00
CR	CR	C=C	HCMM	0.179	3	0.00
HCMM	CR	CR	C=C	0.161	1	0.00
HCMM	CR	CR	C=C	-0.205	2	180.00
HCMM	CR	CR	C=C	0.072	3	0.00
CR	C=C	C=C	CR	-0.202	1	0.00
CR	C=C	C=C	CR	6.000	2	180.00
CR	C=C	C=C	HCMM	6.000	2	180.00
CR	CR	OR	C=O	-0.274	1	0.00
CR	CR	OR	C=O	0.160	3	0.00
C=C	CR	CR	OR	0.150	3	0.00
C=C	C=C	CR	HCMM	0.251	1	0.00
C=C	C=C	CR	HCMM	-0.205	2	180.00
C=C	C=C	CR	HCMM	-0.268	3	0.00
CR	OR	C=O	O=C	0.341	1	0.00
CR	OR	C=O	O=C	3.592	2	180.00
CR	OR	C=O	O=C	-0.468	3	0.00
HCMM	CR	OR	C=O	0.286	1	0.00
HCMM	CR	OR	C=O	-0.152	3	0.00
C=O	CR	CR	OR	-0.340	1	0.00
C=O	CR	CR	OR	-0.015	2	180.00
C=O	CR	OR	HOR	-0.826	1	0.00
C=O	CR	OR	HOR	-0.830	2	180.00
C=O	CR	OR	HOR	0.141	3	0.00
O=C	C=O	CR	OR	-0.198	1	0.00
O=C	C=O	CR	OR	0.365	2	180.00
O=C	C=O	CR	OR	-0.070	3	0.00
OR	C=O	CR	OR	0.224	1	0.00
OR	C=O	CR	OR	0.326	2	180.00
OR	C=O	CR	OR	0.159	3	0.00
OR	CR	CR	OR	0.204	1	0.00
OR	CR	CR	OR	0.699	2	180.00
OR	CR	CR	OR	0.480	3	0.00
OR	C=O	CR	HCMM	-0.312	2	180.00
OR	C=O	CR	HCMM	0.165	3	0.00
O=C	C=O	CR	HCMM	0.330	1	0.00
O=C	C=O	CR	HCMM	-0.704	2	180.00
O=C	C=O	CR	HCMM	0.154	3	0.00
HCMM	CB	CB	HCMM	3.500	2	180.00
HCMM	CR	NRP	HNRP	0.130	3	0.00
HCMM	C=C	C=C	HCMM	6.000	2	180.00
HCMM	C=C	CR	HCMM	-0.262	1	0.00

```
HCMM C=C  CR   HCMM    -0.114  2   180.00
HCMM C=C  CR   HCMM     0.104  3     0.00
```

IMPROPER

```
C5A  C5B  CR   NPYL     3.598  0     0.00
C5B  CR   C5A  C5B      2.879  0     0.00
C5B  C5A  C5B  CB      -0.792  0     0.00
CB   CB   C5B  HCMM     0.864  0     0.00
CB   CB   CB   HCMM     1.079  0     0.00
CR   CB   C5A  CR       0.000  0     0.00
CR   CR   C5A  C=O      0.000  0     0.00
CB   CB   CR   CB       2.879  0     0.00
CB   NC=C CB   CB       3.310  0     0.00
CR   CR   CR   HCMM     0.000  0     0.00
CR   CR   CR   CR       0.000  0     0.00
CR   CR   CR   OR       0.000  0     0.00
NPYL C5A  C5A  HNR     -1.008  0     0.00
CR   CR   C5B  HCMM     0.000  0     0.00
CR   NRP  CR   HCMM     0.000  0     0.00
CB   CB   CB   OR       3.454  0     0.00
NC=C CR   CB   CR      -0.360  0     0.00
CR   CR   NC=C CR       0.000  0     0.00
CR   CR   NC=C HCMM     0.000  0     0.00
CR   CR   CB   CR       0.000  0     0.00
CR   NR   CR   HCMM     0.000  0     0.00
CR   NR   CR   CR       0.000  0     0.00
CR   CR   CR   C=C      0.000  0     0.00
CR   C=C  CR   CR       0.000  0     0.00
C=C  C=C  CR   HCMM     0.936  0     0.00
NR   CR   CR   CR       0.000  0     0.00
CR   CR   CR   C=O      0.000  0     0.00
C=O  OR   CR   O=C     10.147  0     0.00
C=O  O=C  OR   CR      10.147  0     0.00
CR   HCMM CR   HCMM     0.000  0     0.00
CB   C5A  CB   HCMM     0.576  0     0.00
CR   HCMM OR   HCMM     0.000  0     0.00
C=C  CR   C=C  HCMM     0.936  0     0.00
CR   C=C  NR   HCMM     0.000  0     0.00
CR   HCMM C=O  HCMM     0.000  0     0.00
CR   HCMM NC=C HCMM     0.000  0     0.00
NRP  CR   CR   HNRP     0.000  0     0.00
```

NONBONDED nbxmod 5 atom cdiel shift vatom vdistance vswitch –
cutnb 14.0 ctofnb 12.0 ctonnb 10.0 eps 1.0 e14fac 1.0 wmin 1.5

```
C5A     0.000000  -0.050000     2.040000
C5B     0.000000  -0.050000     2.040000
CB      0.000000  -0.070000     1.992400
NPYL    0.000000  -0.090000     1.720000
```

```
CR      0.000000  -0.055000     2.175000   0.000000  -0.010000     1.900000
HCMM    0.000000  -0.022000     1.320000
HNR     0.000000  -0.046000     0.224500
NRP     0.000000  -0.200000     1.850000
OR      0.000000  -0.152100     1.770000
NC=C    0.000000  -0.200000     1.850000
NR      0.000000  -0.200000     1.850000
C=C     0.000000  -0.068000     2.090000
C=O     0.000000  -0.110000     2.000000
O=C     0.000000  -0.120000     1.700000   0.000000  -0.120000     1.400000
HOR     0.000000  -0.046000     0.224500
HNRP    0.000000  -0.046000     0.224500
```

5. Etoposide

PSF file

```
      74 !NATOM
       1 LIG  1    LIG  C    CB    -0.143500      12.0110          0
       2 LIG  1    LIG  C2   CB    -0.143500      12.0110          0
       3 LIG  1    LIG  C3   CB    -0.143500      12.0110          0
       4 LIG  1    LIG  C4   CB    -0.150000      12.0110          0
       5 LIG  1    LIG  C5   CB    -0.150000      12.0110          0
       6 LIG  1    LIG  C6   CB    -0.150000      12.0110          0
       7 LIG  1    LIG  C7   CB    -0.150000      12.0110          0
       8 LIG  1    LIG  C11  CB     0.082500      12.0110          0
       9 LIG  1    LIG  C12  CB     0.082500      12.0110          0
      10 LIG  1    LIG  C13  CB     0.082500      12.0110          0
      11 LIG  1    LIG  C14  CB     0.082500      12.0110          0
      12 LIG  1    LIG  C16  CB     0.082500      12.0110          0
      13 LIG  1    LIG  C8   CR     0.000000      12.0110          0
      14 LIG  1    LIG  C9   CR     0.287000      12.0110          0
      15 LIG  1    LIG  C10  CR     0.000000      12.0110          0
      16 LIG  1    LIG  C15  CR     0.061000      12.0110          0
      17 LIG  1    LIG  C17  CR     0.423500      12.0110          0
      18 LIG  1    LIG  C18  CR     0.280000      12.0110          0
      19 LIG  1    LIG  C19  CR     0.280000      12.0110          0
      20 LIG  1    LIG  C20  CR     0.280000      12.0110          0
      21 LIG  1    LIG  C21  CR     0.280000      12.0110          0
      22 LIG  1    LIG  C22  CR     0.280000      12.0110          0
      23 LIG  1    LIG  C23  CR     0.280000      12.0110          0
      24 LIG  1    LIG  C24  CR     0.280000      12.0110          0
      25 LIG  1    LIG  C25  CR     0.280000      12.0110          0
      26 LIG  1    LIG  H    HOR    0.400000       1.0079          0
      27 LIG  1    LIG  H2   HOR    0.400000       1.0079          0
      28 LIG  1    LIG  H3   HOCC   0.450000       1.0079          0
      29 LIG  1    LIG  C26  CR     0.560000      12.0110          0
      30 LIG  1    LIG  C27  C=O    0.659000      12.0110          0
      31 LIG  1    LIG  O    O=C   -0.570000      15.9994          0
      32 LIG  1    LIG  C28  CR     0.560000      12.0110          0
```

```
33 LIG  1   LIG  O2   OR   -0.430000    15.9994    0
34 LIG  1   LIG  O3   OR   -0.362500    15.9994    0
35 LIG  1   LIG  O4   OR   -0.362500    15.9994    0
36 LIG  1   LIG  O5   OR   -0.560000    15.9994    0
37 LIG  1   LIG  O6   OR   -0.560000    15.9994    0
38 LIG  1   LIG  O7   OR   -0.560000    15.9994    0
39 LIG  1   LIG  C29  CR    0.560000    12.0110    0
40 LIG  1   LIG  O8   OR   -0.362500    15.9994    0
41 LIG  1   LIG  O9   OR   -0.362500    15.9994    0
42 LIG  1   LIG  O10  OR   -0.532500    15.9994    0
43 LIG  1   LIG  O11  OR   -0.560000    15.9994    0
44 LIG  1   LIG  O12  OR   -0.680000    15.9994    0
45 LIG  1   LIG  O13  OR   -0.680000    15.9994    0
46 LIG  1   LIG  H4   HCMM  0.150000     1.0079    0
47 LIG  1   LIG  H5   HCMM  0.150000     1.0079    0
48 LIG  1   LIG  H6   HCMM  0.150000     1.0079    0
49 LIG  1   LIG  H7   HCMM  0.150000     1.0079    0
50 LIG  1   LIG  H8   HCMM  0.000000     1.0079    0
51 LIG  1   LIG  H9   HCMM  0.000000     1.0079    0
52 LIG  1   LIG  H10  HCMM  0.000000     1.0079    0
53 LIG  1   LIG  H11  HCMM  0.000000     1.0079    0
54 LIG  1   LIG  H12  HCMM  0.000000     1.0079    0
55 LIG  1   LIG  H13  HCMM  0.000000     1.0079    0
56 LIG  1   LIG  H14  HCMM  0.000000     1.0079    0
57 LIG  1   LIG  H15  HCMM  0.000000     1.0079    0
58 LIG  1   LIG  H16  HCMM  0.000000     1.0079    0
59 LIG  1   LIG  H17  HCMM  0.000000     1.0079    0
60 LIG  1   LIG  H18  HCMM  0.000000     1.0079    0
61 LIG  1   LIG  H19  HCMM  0.000000     1.0079    0
62 LIG  1   LIG  H20  HCMM  0.000000     1.0079    0
63 LIG  1   LIG  H21  HCMM  0.000000     1.0079    0
64 LIG  1   LIG  H22  HCMM  0.000000     1.0079    0
65 LIG  1   LIG  H23  HCMM  0.000000     1.0079    0
66 LIG  1   LIG  H24  HCMM  0.000000     1.0079    0
67 LIG  1   LIG  H25  HCMM  0.000000     1.0079    0
68 LIG  1   LIG  H26  HCMM  0.000000     1.0079    0
69 LIG  1   LIG  H27  HCMM  0.000000     1.0079    0
70 LIG  1   LIG  H28  HCMM  0.000000     1.0079    0
71 LIG  1   LIG  H29  HCMM  0.000000     1.0079    0
72 LIG  1   LIG  H30  HCMM  0.000000     1.0079    0
73 LIG  1   LIG  H31  HCMM  0.000000     1.0079    0
74 LIG  1   LIG  H32  HCMM  0.000000     1.0079    0
```

BONDS

```
CB   CR    356.737   1.4860
CB   CB    401.068   1.3740
CR   CR    306.432   1.5080
CR   HCMM  342.991   1.0930
CB   OR    404.019   1.3760
```

CR C=O 301.539 1.4920
CR OR 363.214 1.4180
C=O O=C 931.963 1.2220
C=O OR 417.476 1.3550
CB HCMM 381.853 1.0840
HOR OR 560.905 0.9720
HOCC OR 564.143 0.9730

ANGLES
CB CB CB 48.145 119.9770
CB CB CR 57.788 120.4190
CB CB HCMM 40.517 120.5710
CB CB OR 69.663 116.4950
CR CR CR 61.243 109.6080
CR CR HCMM 45.770 110.5490
CB CR CB 70.958 111.3150
CB CR CR 54.406 108.6170
CB CR HCMM 45.122 109.4910
HCMM CR HCMM 37.134 108.8360
CR CR C=O 55.917 107.5170
C=O CR HCMM 46.778 108.3850
CB CR OR 63.186 107.9780
CR CR OR 71.390 108.1330
OR CR HCMM 56.205 108.5770
OR CR OR 83.192 111.3680
CR C=O O=C 67.504 124.4100
CR C=O OR 75.060 109.7160
O=C C=O OR 83.120 124.4250
CR OR C=O 66.424 108.0550
CB OR CR 77.363 102.8460
CR OR CR 86.143 106.9260
CB OR HOCC 52.247 105.4090
CR OR HOR 57.069 106.5030

DIHEDRALS
CB CB CB CB 3.500 2 180.00
CB CB CB HCMM 3.500 2 180.00
CB CB CR CB 0.100 3 0.00
CB CB CR CR 0.225 2 180.00
CB CB CR HCMM -0.210 2 180.00
CB CB CR HCMM 0.196 3 0.00
CB CB CB OR 3.500 2 180.00
CB CR CR CR 0.150 3 0.00
CB CR CR HCMM 0.195 3 0.00
CB CR OR CR 0.100 3 0.00
CB CB CR OR 0.075 3 0.00
CB CR CR C=O 0.150 3 0.00
CB CB OR CR 2.191 2 180.00

CB	CB	CB	CR	3.500	2	180.00
CB	CB	OR	HOCC	1.401	2	180.00
CB	OR	CR	HCMM	0.053	3	0.00
CB	OR	CR	OR	0.100	3	0.00
CR	CR	CR	HCMM	0.320	1	0.00
CR	CR	CR	HCMM	-0.315	2	180.00
CR	CR	CR	HCMM	0.132	3	0.00
CR	CR	C=O	O=C	0.412	1	0.00
CR	CR	C=O	O=C	0.070	2	180.00
CR	CR	C=O	O=C	0.163	3	0.00
CR	CR	C=O	OR	-0.059	1	0.00
CR	CR	C=O	OR	-0.167	2	180.00
CR	CR	C=O	OR	0.101	3	0.00
CR	CR	OR	CR	-0.341	1	0.00
CR	CR	OR	CR	0.378	2	180.00
CR	CR	OR	CR	0.378	3	0.00
CR	CR	OR	C=O	-0.274	1	0.00
CR	CR	OR	C=O	0.160	3	0.00
CR	CB	CB	CR	3.500	2	180.00
CR	CB	CB	HCMM	3.500	2	180.00
CR	CR	CR	CR	0.051	1	0.00
CR	CR	CR	CR	0.341	2	180.00
CR	CR	CR	CR	0.166	3	0.00
CR	CR	CR	OR	-0.344	1	0.00
CR	CR	CR	OR	0.878	2	180.00
CR	CR	CR	OR	0.238	3	0.00
CR	C=O	OR	CR	-0.622	1	0.00
CR	C=O	OR	CR	2.741	2	180.00
CR	C=O	OR	CR	0.182	3	0.00
CR	CR	CR	C=O	0.033	1	0.00
CR	CR	CR	C=O	-0.078	2	180.00
CR	CR	CR	C=O	0.071	3	0.00
CR	OR	CR	OR	0.115	1	0.00
CR	OR	CR	OR	-0.355	2	180.00
CR	OR	CR	OR	0.361	3	0.00
CR	OR	CR	HCMM	0.285	1	0.00
CR	OR	CR	HCMM	0.160	2	180.00
CR	OR	CR	HCMM	0.285	3	0.00
CR	CR	OR	HOR	0.135	2	180.00
CR	CR	OR	HOR	0.118	3	0.00
CR	OR	C=O	O=C	0.341	1	0.00
CR	OR	C=O	O=C	3.592	2	180.00
CR	OR	C=O	O=C	-0.468	3	0.00
HOR	OR	CR	HCMM	0.298	1	0.00
HOR	OR	CR	HCMM	-0.138	2	180.00
HOR	OR	CR	HCMM	0.173	3	0.00
C=O	CR	CR	HCMM	-0.128	1	0.00
C=O	CR	CR	HCMM	0.029	2	180.00

```
C=O  OR   CR   HCMM    0.286  1    0.00
C=O  OR   CR   HCMM   -0.152  3    0.00
O=C  C=O  CR   HCMM    0.330  1    0.00
O=C  C=O  CR   HCMM   -0.704  2  180.00
O=C  C=O  CR   HCMM    0.154  3    0.00
OR   CR   CR   HCMM   -0.327  1    0.00
OR   CR   CR   HCMM    0.536  2  180.00
OR   CR   CR   HCMM    0.140  3    0.00
OR   C=O  CR   HCMM   -0.312  2  180.00
OR   C=O  CR   HCMM    0.165  3    0.00
OR   CB   CB   HCMM    3.500  2  180.00
OR   CB   CB   OR      3.500  2  180.00
OR   CR   CR   OR      0.204  1    0.00
OR   CR   CR   OR      0.699  2  180.00
OR   CR   CR   OR      0.480  3    0.00
HCMM CR   CR   HCMM    0.142  1    0.00
HCMM CR   CR   HCMM   -0.693  2  180.00
HCMM CR   CR   HCMM    0.157  3    0.00
```

IMPROPER

```
CB   CB   CR   CB      2.879  0    0.00
CR   CB   CB   CR      0.000  0    0.00
CR   CR   CB   HCMM    0.000  0    0.00
CB   CB   CB   HCMM    1.079  0    0.00
CB   OR   CB   CB      3.454  0    0.00
CR   C=O  CR   CR      0.000  0    0.00
CR   C=O  CR   HCMM    0.000  0    0.00
CR   CR   CR   CR      0.000  0    0.00
CR   CR   CR   HCMM    0.000  0    0.00
C=O  O=C  CR   OR     10.147  0    0.00
CB   CB   CB   OR      3.454  0    0.00
CR   CR   CB   OR      0.000  0    0.00
CR   CR   OR   OR      0.000  0    0.00
CR   CR   OR   HCMM    0.000  0    0.00
CR   CR   CR   OR      0.000  0    0.00
CR   CR   OR   CR      0.000  0    0.00
CR   OR   OR   CR      0.000  0    0.00
CR   HCMM CR   HCMM    0.000  0    0.00
CR   OR   OR   HCMM    0.000  0    0.00
CR   HCMM OR   HCMM    0.000  0    0.00
CR   OR   CR   HCMM    0.000  0    0.00
```

NONBONDED nbxmod 5 atom cdiel shift vatom vdistance vswitch -
cutnb 14.0 ctofnb 12.0 ctonnb 10.0 eps 1.0 e14fac 1.0 wmin 1.5

```
CB     0.000000  -0.070000    1.992400
CR     0.000000  -0.055000    2.175000   0.000000  -0.010000    1.900000
HOR    0.000000  -0.046000    0.224500
HOCC   0.000000  -0.046000    0.224500
```

C=O	0.000000	-0.110000	2.000000			
O=C	0.000000	-0.120000	1.700000	0.000000	-0.120000	1.400000
OR	0.000000	-0.152100	1.770000			
HCMM	0.000000	-0.022000	1.320000			

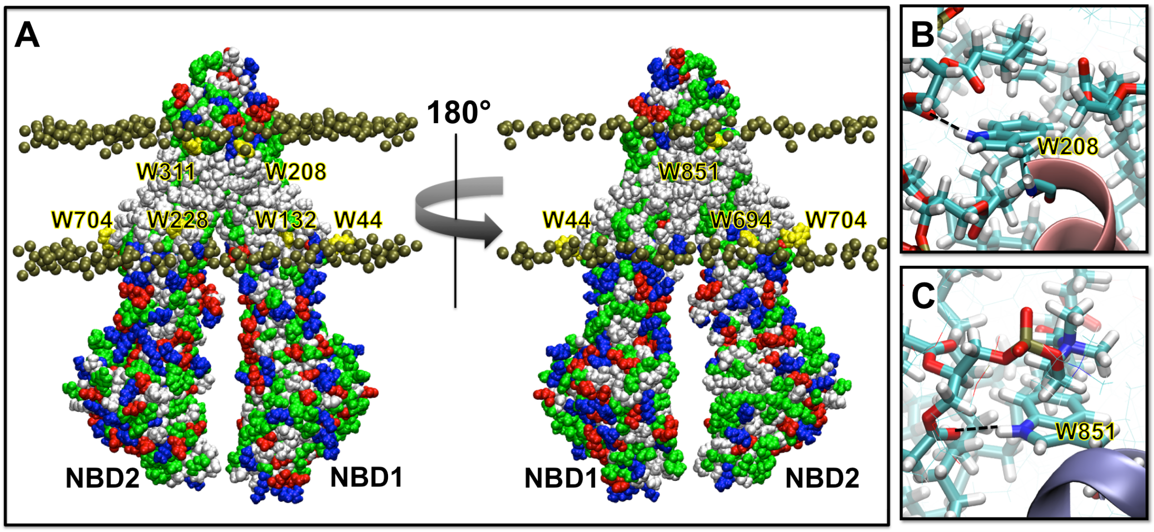

Figure S1. Tryptophan anchors for protein insertion in lipid bilayer. In panel **A**, P atoms of lipids are shown as spheres in tan, protein residues are shown as spheres. The residues are colored based on residue type: non-polar residues are in white, polar-residues are in green, basic residues are in blue and acidic residues are in red. Residues W44, W70, W132, W208, W311, W694, W704 and W851 are in yellow. Panel **B** and **C** show example of H-bond formation between tryptophan and lipid, lipid and Trp residues are drawn in licorice and colored by elements: H in white, N in blue, C in cyan, O in red, P in tan. The NH-O H-bonds between Trp and lipid glyceride are labeled as black dash line.

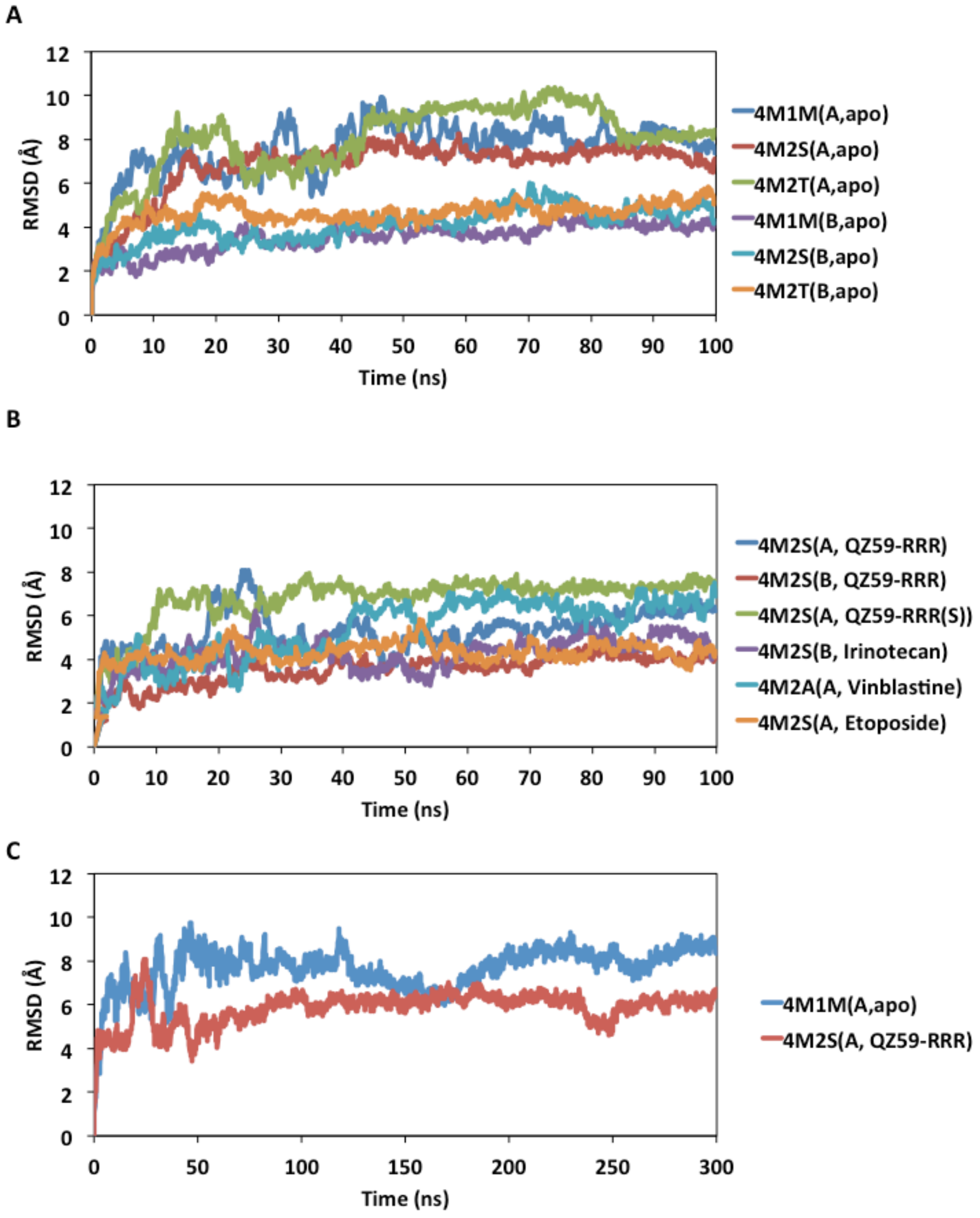

Figure S2. Root-mean-square deviation (RMSD) of protein C_α over time.

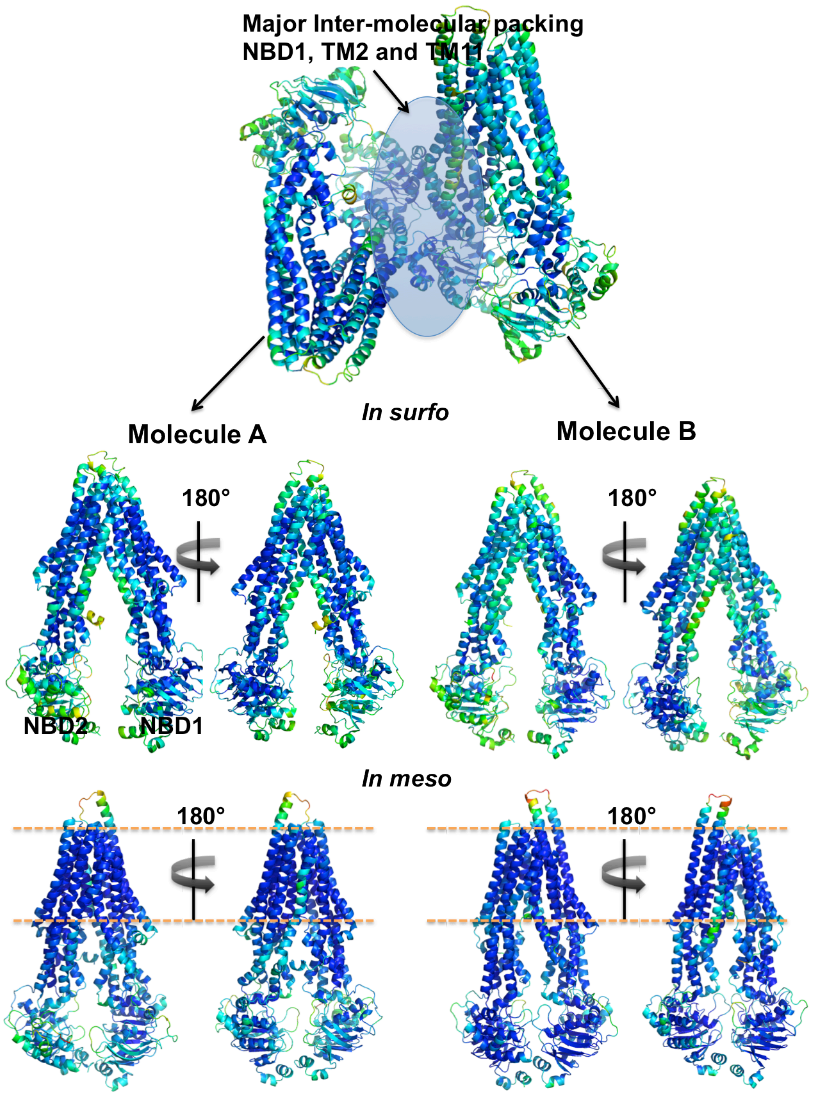

Figure S3. Differences of thermal noises between *in surfo* crystal structure and *in meso* MD simulations. Proteins are shown in cartoon and colored in gradients based on B-factors that high value end in red and low value end in blue. Top panel shows the asymmetric unit in the crystal structure of mouse Pgp (PDB: 4M1M) including two anti-parallel molecules with slightly different conformation: Molecule A and Molecule B. Second panel shows the B-factors for the two molecules in crystal, bottom panel shows the final trajectories at 100 ns for the simulations

of 4M1M(A, apo) and 4M1M(B, apo) and color scales are based on the equilibrated (last 20 ns) RMSFs.

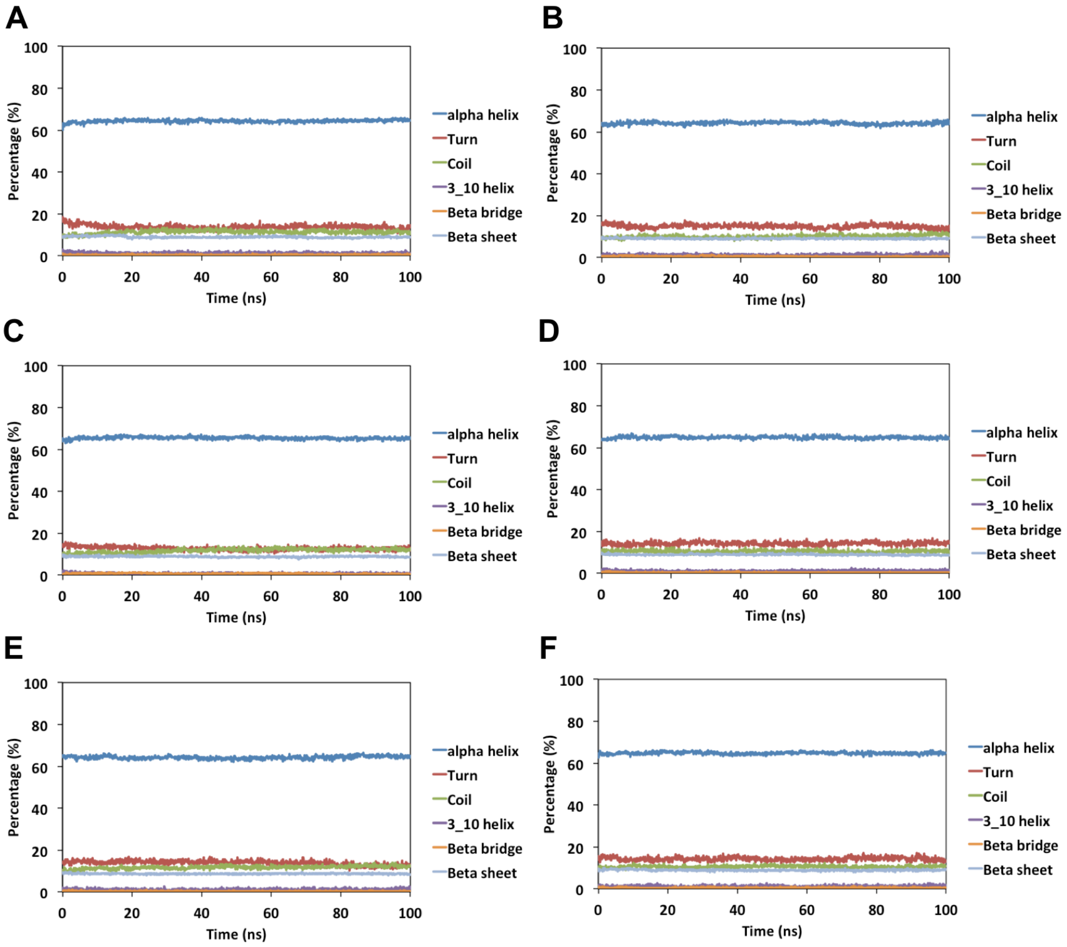

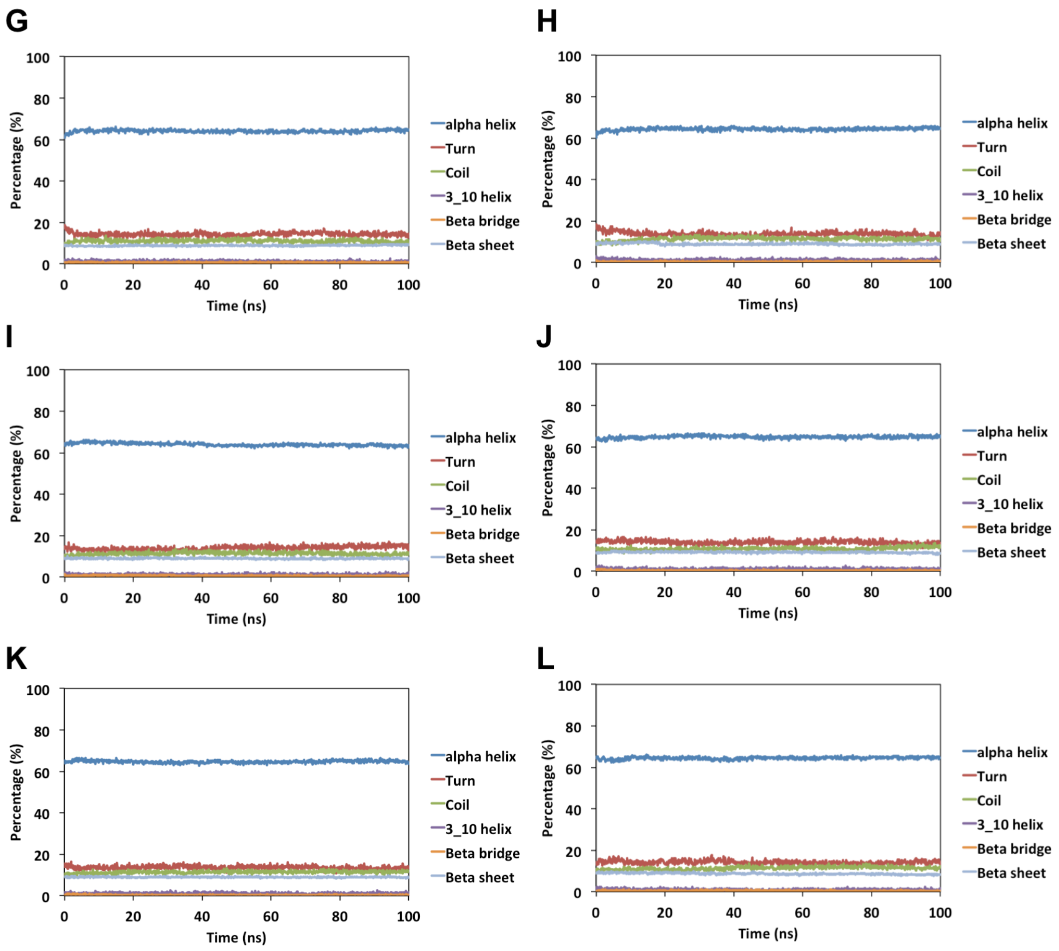

Figure S4. Secondary structure stability over time. Secondary structures were calculated using the STRIDE algorithm. The secondary structure component (alpha helix in blue, turn in red, coil in green, beta sheet in purple, 3_10 helix in orange and beta bridge in light blue) remains consistent during the entire simulation. **A** is 4M1M(A, apo), **B** is 4M2S(A, apo), **C** is 4M2T(A, apo), **D** is 4M1M(B, apo), **E** is 4M2S(B, apo), **F** is 4M2T(B, apo), **G** is 4M2S(A, QZ59-RRR), **H** is 4M2S(B, QZ59-RRR), **I** is 4M2S(A, QZ59-RRR(S)), **J** is 4M2S(B, Irintecan), **K** is 4M2S(A, Vinblastine) and **L** is 4M2S(A, Etoposide).

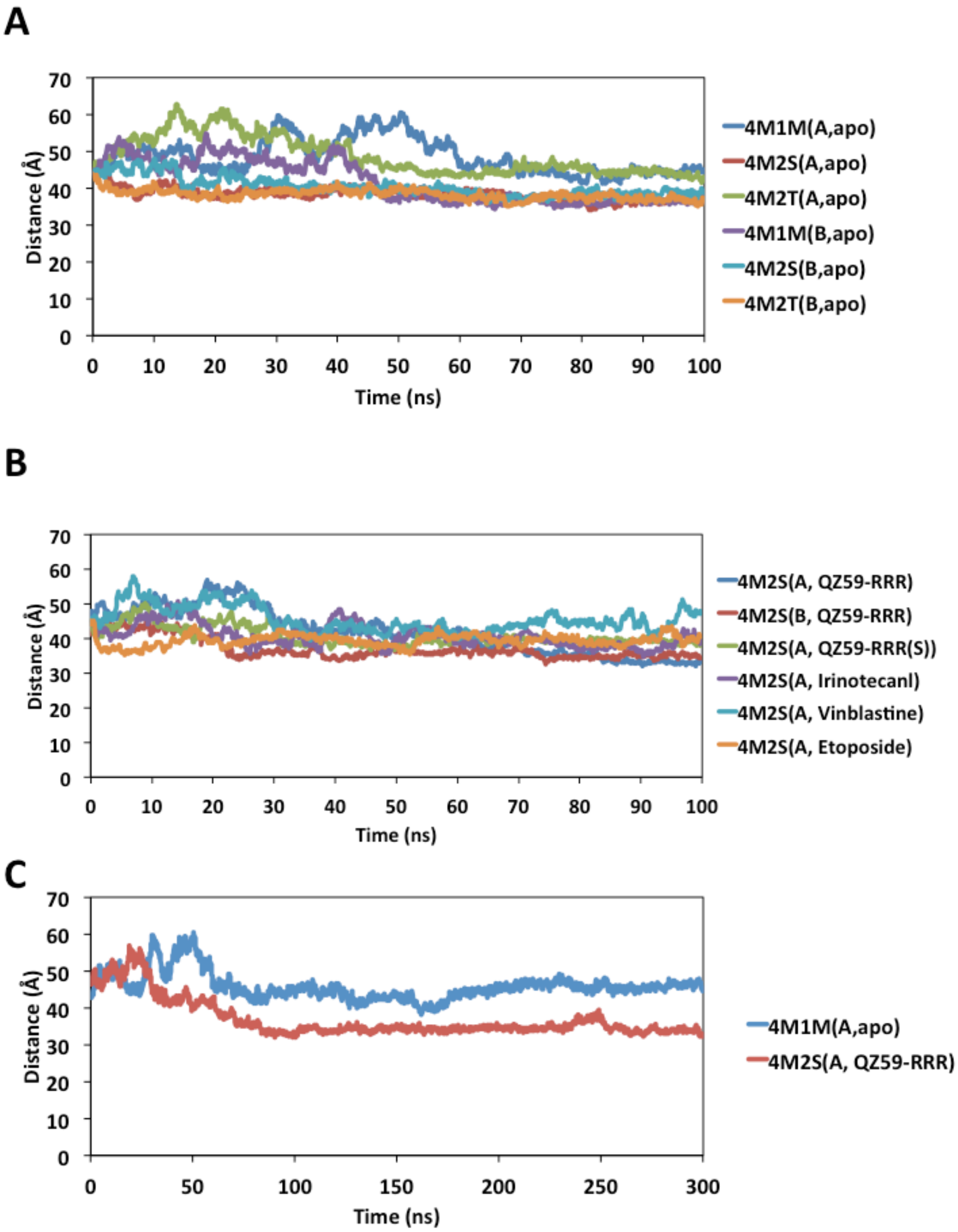

Figure S5. Distances between two NBDs. The distances between two NBDs were measured using the center of mass of each NBD.

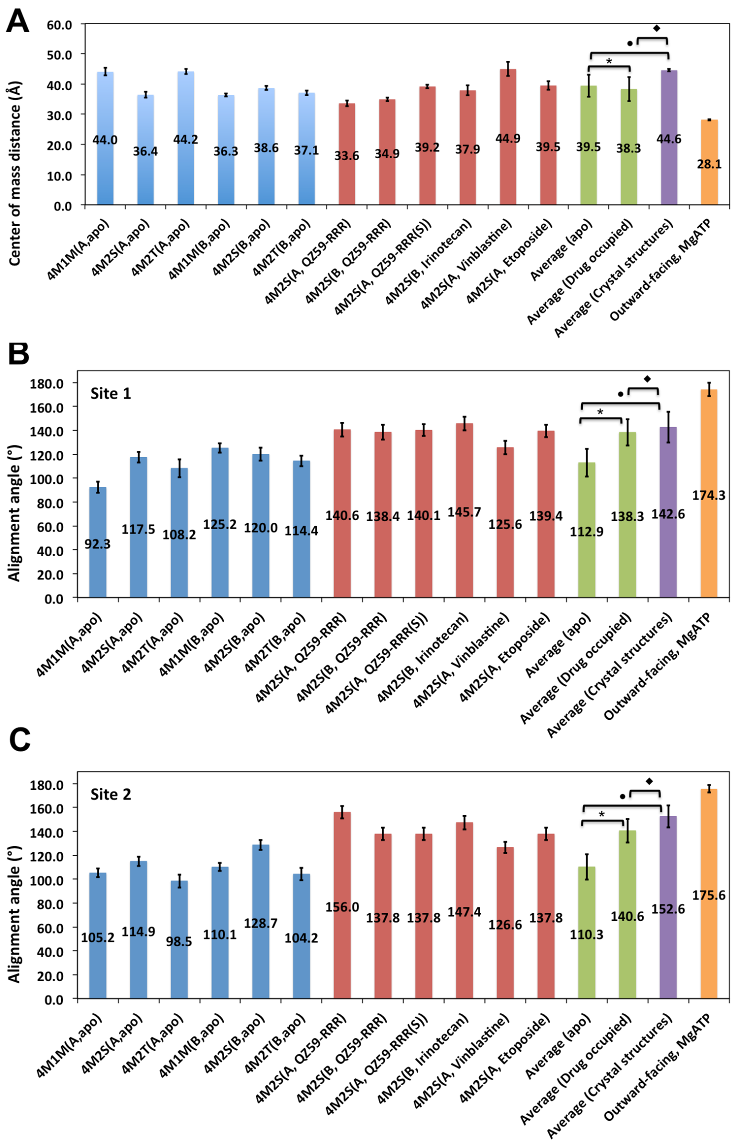

Figure S6. NBDs distances and alignment angles of all simulations. Averages of trajectories over the last 20 ns simulations for six apo-Pgp and six drug-occupied models, six crystal structures(14) and The average of three 200 ns Pgp-MgATP simulations in the outward facing conformation at equilibrium (last 50 ns) were analyzed statistically. Graph **A** shows the distance between the center of mass (COM) of two NBDs. Compared to the crystal structures, both apo-Pgp and drug-Pgp showed significant decrease of NBD COM distances by 5.4 Å (•p=0.007) and 6.3 Å (◆p=0.006). No significant difference (*p=0.334) between apo-Pgp and drug-occupied Pgp. In Graph **B** and **C**, NBD alignment angles are monitored using the angles of C_{α} K429-G1175-L1182 (site1) and K1072-G530-L537 (site2). The alignment angles in drug-occupied Pgp are significantly larger than apo-Pgp at both site 1 (*p=0.003) and site 2 (*p=0.005). Compared to the crystal structure, apo-Pgp species showed significant decreased alignment angles at both site 1 (•p =0.0099) and site 2 (•p =0.0004) while no statistical differences between the drugs occupied Pgp and crystal structures at both site 1 (◆p=0.283) and site 2 (◆p=0.05).

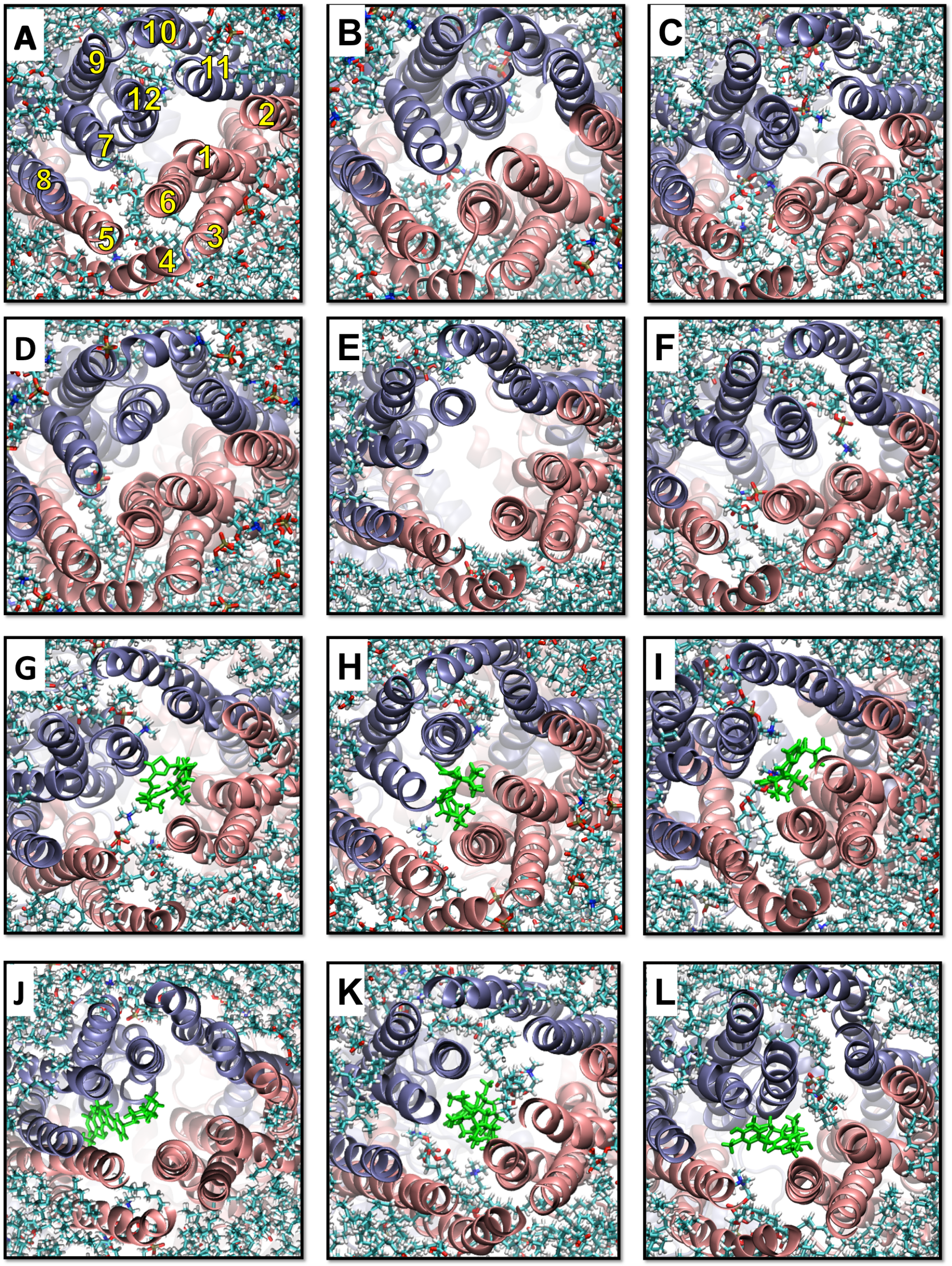

Figure S7. Lipid protrusion at 100 ns for each simulation. The proteins are shown as cartoon with half1 in pink and half2 in ice blue. Lipids are drawn in licorice and colored by elements: H in white, N in blue, C in cyan, O in red, P in tan. Substrates are shown in green licorice. **A** is 4M1M(A, apo), **B** is 4M2S(A, apo), **C** is 4M2T(A, apo), **D** is 4M1M(B, apo), **E** is 4M2S(B, apo), **F** is 4M2T(B, apo), **G** is 4M2S(A, QZ59-RRR), **H** is 4M2S(B, QZ59-RRR), **I** is 4M2S(A, QZ59-RRR(S)), **J** is 4M2S(B, Irintecan), **K** is 4M2S(A, Vinblastine) and **L** is 4M2S(A, Etoposide).

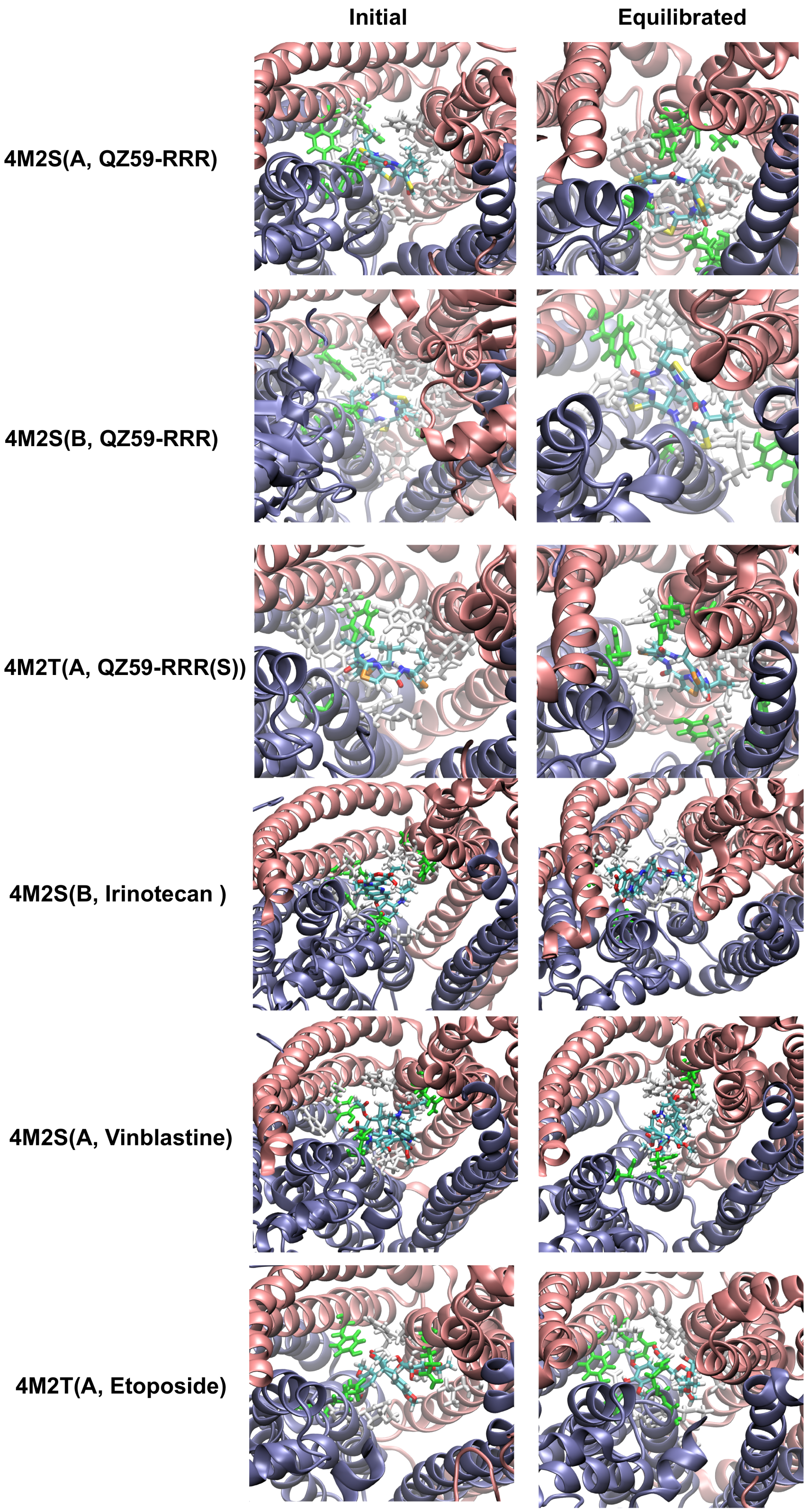

Figure S8. Results of protein drug interactions from crystal structure, docking and simulations. Intracellular views of the protein are provided. The proteins are shown as cartoon with half1 in pink and half2 in ice blue. Initial structures are crystal structure: 4M2S(A, QZ59-RRR), 4M2S(B,

QZ59-RRR) and docking results: 4M2S(A, QZ59-RRR(S)), 4M2S(B, Irinotecan) and 4M2S(A, Vinblastine), 4M2S(A, Etoposide). Equilibrated results are trajectories by the end of each simulation. Drugs are shown in sticks and colored by elements as H in white, N in blue, C in cyan, O in red, S in orange and Se in yellow. Protein residues within 3.0 Å around the drugs were also drawn in sticks and colored by residue types: non-polar residues in white and polar residues in green.

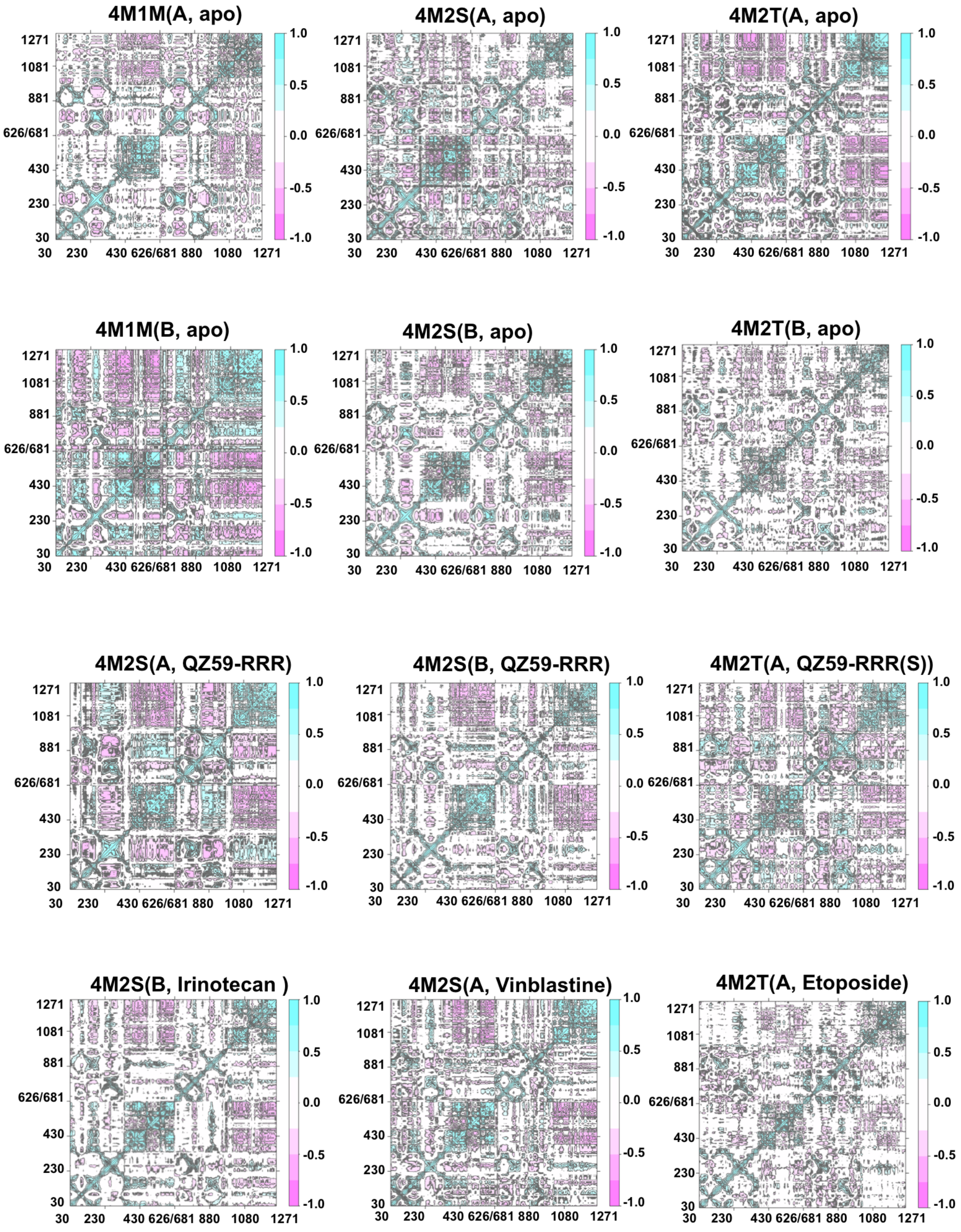

Figure S9. Dynamic cross correlation matrix (DCCM). DCCM was calculated using the trajectories from 100-300 ns simulations of all species. X- and Y-axes are residue numbers representing two halves of Pgp including residue 30-626 and residue 681-1271. Residue 627-680 (the linker region) was not included in the simulations.

Movie S1. Alignment angles in 300 ns simulations of 4M1M (A, apo) and 4M2S (A, QZ59-RRR). The proteins are shown in transparent as cartoon with half1 in pink and half2 in ice blue. Drug like QZ59-RRR is in sticks colored by elements. The alignment angles (as black dash lines) are monitored using the angles of C_α K429-G1173-L1182 (site1, in red spheres) and K1072-G530-L537 (site2, in yellow spheres). All simulations were performed for 300 ns.

Movie S2. Lipid protrusion in 300 ns simulations of 4M1M (A, apo) and 4M2S (A, QZ59-RRR). The proteins are shown as cartoon with half1 in pink and half2 in ice blue. Drug like QZ59-RRR is in sticks colored by elements. Protrusion lipids molecules are drawn in sphere and colored by element (H in white, N in blue, C in cyan, O in red, P in tan). For 4M1M(A, apo), two lipids entered in the front and one lipid entered in the back. For 4M2S(A, QZ59-RRR), one lipid entered from each portal. The P atoms of lipid bilayers are shown in solvent in tan. All simulations were performed for 300 ns.
